# Pleiotropic effect of the proton pump inhibitor esomeprazole leading to suppression of lung inflammation and fibrosis

**DOI:** 10.1186/s12967-015-0614-x

**Published:** 2015-08-01

**Authors:** Yohannes T Ghebremariam, John P Cooke, William Gerhart, Carol Griego, Jeremy B Brower, Melanie Doyle-Eisele, Benjamin C Moeller, Qingtao Zhou, Lawrence Ho, Joao de Andrade, Ganesh Raghu, Leif Peterson, Andreana Rivera, Glenn D Rosen

**Affiliations:** Department of Cardiovascular Sciences, Houston Methodist Research Institute, 6670 Bertner Ave, R10-111, Houston, TX 77030 USA; Department of Cardiovascular Sciences, Houston Methodist Research Institute, 6670 Bertner Ave, R10-211, Houston, TX 77030 USA; Department of Cardiothoracic Surgery, Weill Cornell Medical College of Cornell University, New York, NY USA; Altitude Pharmaceuticals, Inc, San Diego, CA USA; Lovelace Respiratory Research Institute, Albuquerque, NM USA; Department of Respiratory Medicine, Peking University Third Hospital, Beijing, China; Division of Pulmonary Medicine, School of Medicine, Stanford University, Stanford, CA USA; Division of Pulmonary, Allergy and Critical Care Medicine, University of Alabama at Birmingham, Birmingham, AL USA; Division of Pulmonary and Critical Care Medicine, Center for Interstitial Lung Disease (ILD), University of Washington, Seattle, WA USA; Center for Biostatistics, Houston Methodist Research Institute, Houston, TX USA; Department of Pathology and Genomic Medicine, Houston Methodist Research Institute, Houston, TX USA

**Keywords:** Proton pump inhibitors, Inflammation, Oxidative stress, Fibrosis

## Abstract

**Background:**

The beneficial outcome associated with the use of proton pump inhibitors (PPIs) in idiopathic pulmonary fibrosis (IPF) has been reported in retrospective studies. To date, no prospective study has been conducted to confirm these outcomes. In addition, the potential mechanism by which PPIs improve measures of lung function and/or transplant-free survival in IPF has not been elucidated.

**Methods:**

Here, we used biochemical, cell biological and preclinical studies to evaluate regulation of markers associated with inflammation and fibrosis. In our in vitro studies, we exposed primary lung fibroblasts, epithelial and endothelial cells to ionizing radiation or bleomycin; stimuli typically used to induce inflammation and fibrosis. In addition, we cultured lung fibroblasts from IPF patients and studied the effect of esomeprazole on collagen release. Our preclinical study tested efficacy of esomeprazole in a rat model of bleomycin-induced lung injury. Furthermore, we performed retrospective analysis of interstitial lung disease (ILD) databases to examine the effect of PPIs on transplant-free survival.

**Results:**

The cell culture studies revealed that esomeprazole controls inflammation by suppressing the expression of pro-inflammatory molecules including vascular cell adhesion molecule-1, inducible nitric oxide synthase, tumor necrosis factor-alpha (TNF-α) and interleukins (IL-1β and IL-6). The antioxidant effect is associated with strong induction of the stress-inducible cytoprotective protein heme oxygenase-1 (HO1) and the antifibrotic effect is associated with potent inhibition of fibroblast proliferation as well as downregulation of profibrotic proteins including receptors for transforming growth factor β (TGFβ), fibronectin and matrix metalloproteinases (MMPs). Furthermore, esomeprazole showed robust effect in mitigating the inflammatory and fibrotic responses in a murine model of acute lung injury. Finally, retrospective analysis of two ILD databases was performed to assess the effect of PPIs on transplant-free survival in IPF patients. Intriguingly, this data demonstrated that IPF patients on PPIs had prolonged survival over controls (median survival of 3.4 vs 2 years).

**Conclusions:**

Overall, these data indicate the possibility that PPIs may have protective function in IPF by directly modulating the disease process and suggest that they may have other clinical utility in the treatment of extra-intestinal diseases characterized by inflammatory and/or fibrotic phases.

**Electronic supplementary material:**

The online version of this article (doi:10.1186/s12967-015-0614-x) contains supplementary material, which is available to authorized users.

## Background

Idiopathic pulmonary fibrosis (IPF) is a fibrosing lung disease of unknown etiology that causes progressive loss of lung function. It has an incidence of 93.7 cases per 100,000 and prevalence of 494.5 cases per 100,000 [1]. Most patients are over 60 years old, but patients with familial IPF may present earlier [2–4]. IPF has a median survival of only 3–4 years from the time of diagnosis [1]. Recently FDA approved drugs, pirfenidone and nintedanib, only slow the disease progression [5, 6] and development of more effective therapies are hampered by an incomplete understanding of the factors involved in the disease pathogenesis.

Recently, a number of retrospective or prospective case series studies that reviewed interstitial lung disease databases have reported that IPF patients placed on anti-acid therapy (mainly on proton pump inhibitors; PPIs, in comparison to Histamine H_2_-inhibitors; H_2_Is) appear to have improved outcomes in measures of lung function and overall health including longer period of lung transplant-free survival, reduced hospitalization for pulmonary-related illnesses and significantly reduced episodes of acute exacerbations [7–9]. However, given the high prevalence of gastric reflux in IPF patients [10], it is unclear whether the presumed beneficial effect of PPIs is due primarily to the action of reducing gastric acidity and consequently suppressing apparent microaspiration to pulmonary parenchyma or due to potential regulation of other biological processes involved in IPF pathogenesis.

**Fig. 1:**
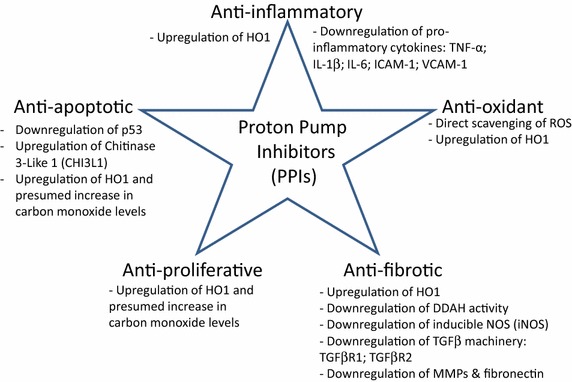
Overview of pleiotropic effect of proton pump inhibitors (PPIs). The PPIs modulate inflammation, oxidative stress, fibrosis, cell proliferation and survival by regulating signaling pathways that are involved in these processes.

PPIs are a class of drug that share a benzimidazole compound as a common core structure and are known to possess other biological activities apart from suppression of proton pumps which are mainly expressed by acid-secreting parietal cells of the stomach [11]. In vitro and in vivo studies have shown that PPIs have antioxidant and anti-inflammatory functions in various cell types including immune, vascular endothelial and epithelial cells [12, 13]. The antioxidant property of the PPIs is reported to be due to direct scavenging of reactive oxygen species (ROS) and induction of the stress-inducible protein heme oxygenase-1 (HO1) [12, 14]. Meanwhile, the effect on inflammation was attributed to regulation of neutrophil chemotaxis and phagocytosis [15, 16], attenuation of free radical production by immune cells [17–19] and downregulation of pro-inflammatory/profibrotic cytokines [13, 20] as well as inhibition of interaction between inflammatory and vascular cells [21] (Fig. 1).

Recently, we reported [22] that the PPIs regulate the nitric oxide (NO) synthase (NOS) pathway by inhibiting dimethylarginine dimethylaminohydrolase (DDAH); an enzyme that is ubiquitously expressed in various segments of the lungs including the endothelium, bronchi and alveoli, in one of two isoforms [23]. In IPF, it has been reported that the NOS/DDAH pathway is upregulated [24]. Moreover, IPF patients show increased markers of nitrosative stress, including significantly higher levels of plasma nitrite [24, 25]. Genetic manipulation or pharmacological treatment of mice that show IPF-like lung injury with inhibitors of NOS or DDAH improve lung compliance [24, 26].

Overall, simultaneous regulation of inflammation as well as oxidative and nitrosative stress by the PPIs led us to develop the following hypotheses: (1) PPIs reduce the production of pro-inflammatory cytokines by lung epithelial and vascular endothelial cells exposed to inflammatory stimuli; (2) PPIs regulate markers of fibrosis including the production of soluble collagen by lung fibroblasts isolated from IPF patients; (3) PPIs attenuate inflammation and fibrosis in an animal model of acute lung injury; and (4) IPF patients receiving PPIs would have better clinical outcomes compared to IPF patients not receiving PPIs.

Accordingly, we conducted in vitro and in vivo experiments to evaluate the ability of a prototype PPI, esomeprazole, in regulating markers of inflammation, apoptosis, oxidative stress and fibrosis in primary lung cell types including fibroblasts, epithelial as well as endothelial cells. In vivo, we assessed the efficacy of esomeprazole in attenuating lung inflammation and fibrosis in a rat model of bleomycin-induced lung injury. Clinically, we performed retrospective study to assess if there is a correlation between PPI use and prolonged lung transplant-free survival in IPF patients including these who have had no symptoms of gastric reflux; a condition for which the PPIs are mainly prescribed.

## Methods

### Cell proliferation assay

Lung fibroblasts were isolated under consent from IPF patients undergoing transplantation as we described previously [27]. Characterization of the cells is described in the Additional file 1 of this manuscript. For the proliferation assay, 3 × 10^3^ cells/well were seeded in a 96-well plate and incubated overnight at 37°C/5%CO_2_. The next day, the cells were synchronized by serum starvation for 2 h followed by 22 h low serum (0.1% fetal bovine serum, FBS) treatment. On day 3, the cells were stimulated with 10% FBS containing media in the presence of esomeprazole or vehicle and were further cultured under this condition for 24 h. Furthermore, the cells were incubated with 5-bromo-2-deoxyuridine (BrdU; 20 µL of 1:500 dilution) for 24 h to assess the effect of esomeprazole on proliferation. Finally, the incorporation of BrdU into newly synthesized DNA of proliferating cells was detected immunochemically using an antibody directed against BrdU using a BrdU Cell Proliferation Assay kit (Millipore). Similarly, the effect of esomeprazole on the proliferation of primary lung epithelial cells (Lonza) was assessed.

### Exposure of primary lung cells to bleomycin

IPF lung-derived or normal lung fibroblasts as well as normal primary lung epithelial and endothelial cells (Lonza) were cultured and expanded using standard cell culture techniques. In this study, the cells were treated with esomeprazole or vehicle for 24 h in the presence or absence of bleomycin (Sigma; at 25 µg/mL final concentration). Subsequently, the cells were harvested and total RNA was extracted using the PerfectPure RNA Cell and Tissue kit (5 PRIME). Next, the concentration and quality of the RNA were validated using a Nanodrop (Tecan) and 2 µg of RNA was reverse transcribed using the High Capacity RNA-to-cDNA Kit (Applied Biosystems). The resulting cDNA was used for gene expression study by quantitative RT-PCR. Quantitative RT-PCR (qRT-PCR) was performed using standard TaqMan gene expression assay using proprietary “best coverage” primer/probe sets (Life Technologies) as described below.

### Exposure of primary lung epithelial cells to ionizing radiation in a 3D culture system

For ionizing radiation experiments, primary lung epithelial cells were cultured in a three-dimensional (3D) culture system. In brief, standard T75 flasks were coated with MaxGel (Sigma) extracellular matrix (ECM; a human basement membrane extract which provides tissue-like microenvironment due to its composition of collagen, fibronectin, laminin, elastin and other proteoglycans) for 4 h at 37°C/5%CO_2_. The solution was aspirated prior to allowing the flasks to air-dry for 30 min at room temperature. Subsequently, primary human bronchial epithelial cells (Lonza) were suspended in epithelial cell growth media (BEGM) and seeded in the pre-coated 3D flasks. When the cells reached 60% confluency, they were treated with esomeprazole or vehicle for 24 h prior to irradiation. The cells were X-ray irradiated by exposure to 6 Gray (6 Gy) of continuous ionizing radiation (RS-2000 Biological System; Rad Source Technologies) applied at a rate of 2 Gy/min. Subsequently, the irradiated and control cells were incubated at 37°C/5%CO_2_ for additional 6 h prior to harvesting and RNA extraction as described above.

### Gene expression study

For gene expression study, cDNA was generated as described above and was used for real-time RT-PCR to compare the effect of esomeprazole on the mRNA expression of HO1, tumor necrosis factor alpha (TNF-α), interleukins (IL-1β and IL-6), p53, adhesion molecules (VCAM-1 and ICAM-1), TGFβ and its receptors (TGFβR1 and TGFβR2), matrix metalloproteinases (MMPs), collagen type 1 (COL1A1), fibronectin 1 (FN1), Chitinase 3-like 1 (CHI3L1) and inducible NOS (iNOS). A QuantStudio 12 K Flex Real-Time PCR System (Life Technologies) was used for the analyses. Each reaction contained 10 µL of TaqMan Universal PCR Master mix (2X), 1 µL of TaqMan assay containing primers and MGB probe mix (20X) and 3 µL of cDNA in 20 µL final volume. The reaction was carried out in a 96-well plate under the following condition: incubation at 50°C for 2 min; denaturation at 95°C for 10 min followed by 95°C for 15 s and finally annealing and extension at 60°C for 2 min for 34 cycles in total. The data was analyzed using the QuantStudio gene expression software and fold changes in mRNA expression were calculated by standardizing to β-actin internal control.

### In vivo study of bleomycin-induced lung inflammation and fibrosis

We conducted a 28-day study using male Fischer rats (F344 strain) to assess the efficacy of esomeprazole in inhibiting or attenuating the progression of bleomycin sulfate-induced lung fibrosis using an intra-tracheal (IT) instillation model. The study was conducted at Lovelace Respiratory Research Institute (LRRI) and the experimental design (shown in Table 1) consisted of 6 randomized groups receiving bleomycin sulfate (BS) and 1 group receiving saline by intra-tracheal instillation on study day 0. In brief, the animals were anesthetized using 4–5% isoflurane in oxygen until a deep plane of anesthesia was achieved. Next, normal saline or BS dissolved in normal saline (250–340 µL based on body weight) was administered to each animal according to their grouping. Following instillation and brief recovery, animals that received normal saline (Group 1) received vehicle (10% ethanol) once daily (QD) by oral gavage (PO) on days 10–28. BS control animals (Group 2) received vehicle (QD, PO) on days 10–28. Groups 3–6 received esomeprazole at 30 or 300 mg/kg (QD, PO prepared in 10% ethanol) on Days 2–28 (prophylactic model) or 10–28 (therapeutic model). Group 7 received the control drug pirfenidone [28] at 100 mg/kg by PO, twice daily (BID) on days 10–28. During the course of the study, blood samples were collected for biochemical and pharmacokinetic study as described below. On day 28, shortly after the final drug dose, the animals were euthanized using pentobarbital solution. At necropsy, terminal blood samples were collected by cardiac puncture, processed to plasma, and stored frozen for bioanalytical and biochemical studies. In addition, bronchoalveolar lavage (BAL) was performed and BAL fluid (BALF) collected for soluble collagen analysis as described below. Furthermore, the left lung lobes were fixed for DNA fragmentation (TUNEL) assay and for histological analyses including H&E and Sirius Red collagen staining as described below. The right caudal lung lobes were used for hydroxyproline assay as described below and the right lung lobes were individually flash frozen and subsequently used for microarray study as described below.

**Table 1:** Experimental design of bleomycin sulfate (BS)-induced lung fibrosis in a 28-day rat model

Group ID	Exposure	N	Bleomycin dose (mg/kg), Route	Eso dose (mg/kg)	Route	Dosing days	Necropsy day
1	Saline-vehicle control	6	0, IT	Vehicle	PO	10–28	28
2	BS control	10	~4.0, IT	Vehicle	PO	10–28	28
3	BS + eso low therapeutic	10	~4.0, IT	30	PO	10–28	28
4	BS + eso high therapeutic	10	~4.0, IT	300	PO	10–28	28
5	BS + eso low prophylactic	15	~4.0, IT	30	PO	2–28	28
6	BS + eso high prophylactic	15	~4.0, IT	300	PO	2–28	28
7	BS + pirfenidone	10	~4.0, IT	100	PO, BID	10–28	28

Initially, the groups were exposed to normal saline or BS intra-tracheally (IT) and then received vehicle, esomeprazole (prophylactically or therapeutically) or therapeutic pirfenidone orally (PO) for the indicated course.

*N* number of animals, *BID* twice daily, *Eso* esomeprazole.

### Pharmacokinetics of esomeprazole

Five animals each from the low-dose and high-dose of prophylactic esomeprazole were used to determine the concentration of esomeprazole in plasma and lung tissue. In brief, blood was collected prior to dosing on day 0, 1 h after dosing on day 15, 1 h after dosing on day 20, and again at sacrifice (day 28). Meanwhile, pharmacokinetic (PK) study was carried out on day 5 by collecting blood at 0.5, 1, 2, and 3 h post dosing. Esomeprazole in plasma was extracted using a protein precipitation procedure. Briefly, the plasma was thawed from storage at −80°C and aliquoted in 100 µL. To this, 50 µL of acetonitrile or spiking solutions in acetonitrile were first added followed by the addition of 300 µL of acetonitrile containing 10 ng/mL of omeprazole-d3 (internal standard). The samples were mixed for 10 s and then centrifuged at 13,000 rpm for 5 min. The supernatant was transferred to labeled autosampler vials with inserts, and the drug concentration was analyzed by liquid chromatography-mass spectrometry (LC–MS) methods developed at Lovelace Respiratory Research Institute (LRRI) and based on published protocol [29]. Concentration versus time values in plasma samples were used to determine PK parameters of esomeprazole including half-life (T_1/2_), peak concentration (C_max_), time to peak concentration (T_max_) and area under the concentration/time curve (AUC). Similarly, esomeprazole in lung tissue was extracted using a protein precipitation procedure. Briefly, the lungs were thawed from storage at −80°C and 100 mg each was homogenized in 1 mL of Dulbecco’s phosphate buffered saline (PBS). From the homogenate, 100 µL of supernatant was aliquoted and processed for determination of tissue esomeprazole concentration as described for the plasma samples above.

### Soluble collagen and hydroxyproline assays

For the quantification of soluble collagen in BALF, the right lungs were lavaged twice with 3 mL of PBS. The lavagates were pooled together for each group and 200 µL of supernatant each was assayed for soluble collagen by colorimetric Sircol assay following the manufacturer’s (BioColor) recommended protocol. Similarly, the right caudal lung lobe was homogenized and analyzed for tissue hydroxyproline content by colorimetric assay. Finally, the amount of collagen in the BALF and lung tissue samples was estimated from standard curve and was expressed as µg collagen per 200 µL BALF and µg collagen per lung lobe respectively.

### ELISA assays

The concentration of IL-1β, MMP7, CHI3L1, bilirubin, ADMA and NO in rat plasma was determined using respective ELISA-based biochemical assays as per the recommendations of the respective commercial purveyors. Respective standard curves were used to estimate the concentration of each of the analytes and the Mean values were used for comparison among the groups.

### Histopathology and immunofluorescence study

For this study, left lung lobes were fixed in 10% neutral buffered formalin (NBF) and then processed to slides for immunohistochemistry. In brief, the tissues were trimmed beginning at a random start point approximately 3–5 mm from the cranial end of the lobe. Fixed lungs were cut transversely each 3–4 mm and every sectioned tissue was submitted for histology. Tissues were paraffin embedded and sectioned at approximately 4 µm thickness to produce two serial sections for histopathological analyses. One section was stained with Hematoxylin and Eosin (H&E) in order to assess inflammation and overall tissue architecture and the other was stained with Sirius Red (SR) to examine collagen deposition and fibrotic changes. H&E slides and corresponding SR slides were microscopically examined together and graded subjectively according to their degree of inflammation and fibrosis respectively. In brief, a pathologist graded the lesions in a semi-quantitative fashion on a scale of 1–4 (1 = minimal, 2 = mild, 3 = moderate, 4 = severe).

For the immunohistochemical staining of rat lung tissue for alpha-smooth muscle actin (α-SMA; Sigma) and Collagen 1 (Col1A1; Sigma), we used standard staining protocol. In brief, paraffin embedded sections were mounted into slides (5 µm thickness) and incubated overnight in a 60°C oven. Next, the slides were allowed to cool prior to deparaffinization and rehydration. Subsequently, the antigen was retrieved by boiling the slides in 10 mM sodium citrate solution (pH 6.0) and non-specificity was blocked with 2.5% horse serum for 30 min at room temperature (RT). Subsequently, the slides were incubated with their respective primary antibodies: α-SMA (1:2,000), Col1A1 (1:2,000) for 1 h at RT. The next day, the antibodies were washed off and the slides were incubated with biotinylated secondary antibodies for 30 min at RT in moist chamber. Finally, the slides were incubated in streptavidin-HRP solution for 30 min prior to adding DAB substrate, counterstaining with Hematoxylin and mounting. Multiple non-overlapping microscopic fields were scanned and reviewed by a pathologist in a blinded fashion.

### TUNEL assay

The left lung lobe was processed to slides, stained for fragmented DNA (TUNEL assay) and analyzed at LRRI’s Pulmonary Fibrosis Laboratory to determine the ratio of apoptotic cells. In addition, double immunofluorescence staining was performed for the pro-apoptotic protein p53 (Invitrogen) and the epithelial cell marker prosurfactant protein C (proSP-C; Millipore) in order to delineate the degree of apoptosis in the epithelial cells subpopulation. In brief, slide-mounted lung sections were simultaneously stained with both primary antibodies (1:200 diluted mouse anti-p53 and 1:800 diluted rabbit anti-proSP-C) and then with their respective secondary antibodies conjugated to different fluorophores for differential analysis. The slides were mounted in a mounting media containing DAPI (nuclear stain) and examined for colocalization of the two proteins to mark the degree of epithelial cell death.

### Lung tissue microarray study

For this study, total RNA was extracted from lung tissue homogenates using the isolation technique described above. The quality of the RNA including the concentration and integrity was checked using a bioanalyzer 2100 (Agilent Technologies) at the Baylor College of Medicine, Genomic and RNA Profiling Core. Subsequently, RNA samples were subjected to GeneChip Rat Exon ST 1.0 Array for comprehensive analysis of the rat lung genome. In brief, the RNA (100 ng each) was first converted to first-strand cDNA and then to second-strand prior to overnight amplification of the cRNA. The next day, the cRNA (15 µg) was purified and used for single-strand cDNA (ss-cDNA) synthesis. Next, the template RNA was removed and the ss-cDNA (5.5 µg) was purified prior to being fragmented and biotin conjugated for hybridization. Subsequently, 200 µL hybridization cocktail containing Affymetrix spike-in controls and the conjugated cDNA was loaded onto a GeneChip^®^ Rat Exon 1.0 ST array. The arrays were hybridized for 17 h at 45°C, with rotation at 60 rpm on a GeneChip^®^ Hybridization Oven 640. The arrays were then washed and stained with a Streptavidin, R-phycoerythrin conjugate on a GeneChip^®^ Fluidics Station 450. Signal amplification was assessed using biotinylated antistreptavidin. The stained arrays were scanned on an Affymetrix GeneChip^®^ Scanner 3000. The images were analyzed and quality control metrics recorded using Affymetrix Command Console software version 4.0.0. Finally, the transcript expression data was clustered into signaling pathways and presented in heatmap format for comparison.

### IPF patient population, demographics and clinical data

Two hundred fifteen (215) patients from the Stanford University and the University of Alabama at Birmingham (UAB) ILD databases, diagnosed with IPF according to evidence-based guidelines [30] were studied. Patients were analyzed in the PPI treatment group if they were on any PPI for at least 12 months. Survival time was defined as time to either death or lung transplantation. Patients were excluded if: (1) they were lost to follow-up before 12 months; (2) pulmonary function tests (PFTs) were not available; (3) FEV1/FVC was ≤0.70; and (4) PPI therapy lasted less than 12 months for reasons other than lung transplantation or death.

### Statistical analysis

For the in vitro cell culture studies, Mean value ± standard error mean (SEM) of each data is presented unless stated otherwise. Multiple groups were compared using one-way analysis of variance (ANOVA) followed by Bonferroni posttest correction. Two groups were compared by student’s *t* test using GraphPad Prism 5 software (La Jolla, CA, USA).

For the in vivo preclinical study, Means and standard deviations (SD) were calculated for collagen and hydroxyproline measurements. ANOVA was performed to evaluate the effect of bleomycin sulfate and esomeprazole or pirfenidone treatment, to compare treatment groups for soluble collagen, hydroxyproline, and other endpoints. Where there was a significant treatment effect (p < 0.05), Dunnett’s multiple comparison was performed to assess differences between treated and control groups.

For the microarray study, a one-way ANOVA based on the method of Eisenhart [31] was used for treatment effect (prophylactic, therapeutic and vehicle). Fisher’s least significant difference was used with contrasts [32] for pairwise group comparisons: prophylactic vs. therapeutic, prophylactic vs. vehicle, and therapeutic vs. vehicle.

The clinical data for survival was analyzed using Kaplan–Meier. Data was presented as statistically significant when the p value was less than 0.05 (p < 0.05). A Cox proportional hazard model was used to identify predictors of survival time, i.e., time to transplant or death.

### Study approval

The animal study was reviewed and approved by Lovelace Respiratory Research Institute (LRRI)’s vertebrate animal use research committee (IACUC approval # FY14-084).

The isolation of lung fibroblasts from IPF patients was performed under consent following review and approval by Stanford University’s Institutional Review Board (IRB) (approval # 18891).

All other reagents used in this study are from commercial sources.

## Results

### Esomeprazole inhibits the proliferation of primary lung cells in vitro

**Fig. 2:**
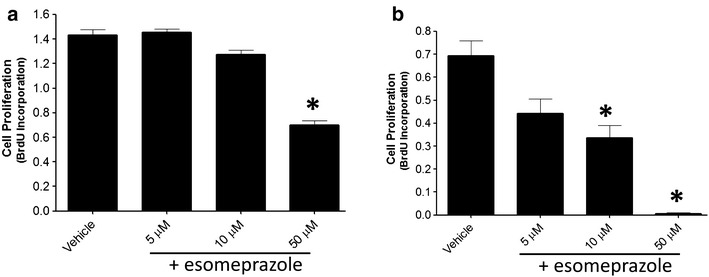
The effect of esomeprazole on **a** lung fibroblast and **b** alveolar epithelial cell proliferation. Cells were synchronized and then serum-stimulated to induce proliferation in the presence of vehicle or esomeprazole (5–50 µM). Incorporation of BrdU into newly synthesized DNA was quantified spectrophotometrically. Data is Mean ± SEM from duplicate experiments. *p < 0.05 compared to vehicle. *BrdU* Bromodeoxyuridine.

Hyperplasia of alveolar epithelial (ATII) cells, although inconclusive, is reported to be pathologically involved in lung fibrosis through the process of epithelial-to-mesenchymal transition (EMT) [33, 34]; by serving as precursor cells for fibroblast-driven fibrosis. In addition, over-proliferation of fibroblasts contributes to pathological deposition of extracellular matrix in the lungs [35]. In this study, we found that esomeprazole dose-dependently attenuated serum-induced proliferation of both lung fibroblasts (by about 50% at 50 µM; p < 0.05) and epithelial cells (by over 90% at 50 µM; p < 0.05), as demonstrated by reduced BrdU incorporation into newly synthesized DNA (Fig. 2). These effects of esomeprazole occurred at doses that were not associated with cytotoxicity. Specifically, we treated lung fibroblasts and epithelial cells with increasing concentration of esomeprazole for 24 h and studied the release of lactate dehydrogenase (LDH) into the conditioned media. Treatment with esomeprazole at concentrations significantly higher than used in the proliferation assay was not associated with cytotoxicity (Additional file 1: Figure S1).

### Esomeprazole attenuates elaboration of inflammatory response to bleomycin and radiation in vitro

**Fig. 3:**
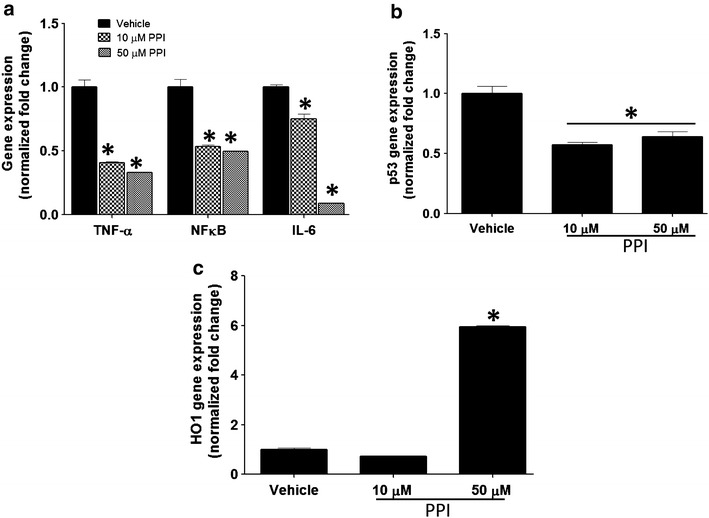
The PPI esomeprazole regulates ionizing radiation-induced changes in gene expression in primary lung epithelial cells cultured in 3D matrix. In **a** inhibition of the spike in the pro-inflammatory cytokines TNF-α, NFκB and IL-6 is shown. **b** shows downregulation of the proapoptotic protein p53 and **c** shows the upregulation of the antioxidant gene HO1 by esomeprazole. Data is Mean ± SEM from duplicate experiments. *p < 0.05 compared to vehicle.

**Fig. 4:**
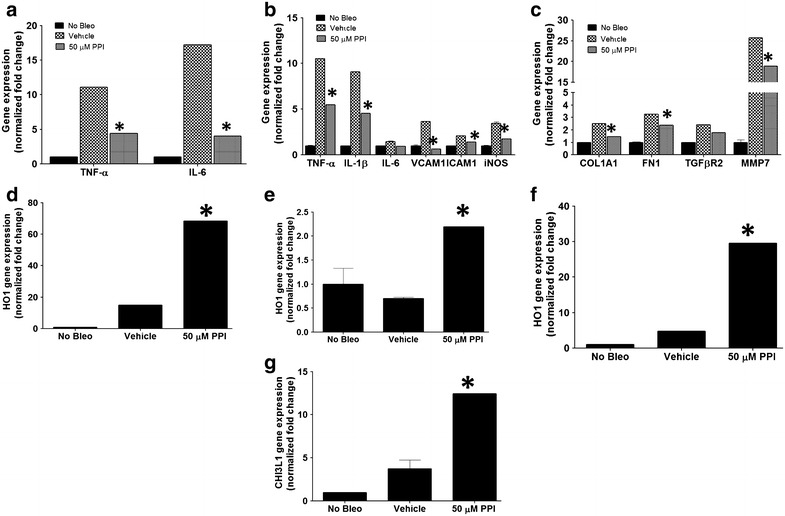
Esomeprazole regulates bleomycin-induced changes in gene expression in various lung cell types. In **a**–**c** suppression of pro-inflammatory/pro-fibrotic markers in primary epithelial (**a**), endothelial (**b**) and fibroblasts (**c**) is shown. **d**–**f** demonstrates increased expression of the cytoprotective enzyme HO1 by esomeprazole in each of these cell types. **g** shows upregulation of the lung repair associated gene chitinase-3-like 1 (CHI3L1) in primary lung epithelial cells. Data is Mean ± SEM from duplicate experiments. *p < 0.05 compared to vehicle.

A typical cellular response to bleomycin or ionizing radiation is a burst in markers of inflammation [36, 37]. Interestingly, pre-incubation of primary lung epithelial cells with esomeprazole prior to exposure to ionizing radiation significantly (by about 50%; p < 0.05) suppressed the expression of pro-inflammatory markers including TNF-α, IL-6 and nuclear factor kappa B (NFκB) (Fig. 3a). Moreover, esomeprazole downregulated the expression of p53 (Fig. 3b) and upregulated the anti-inflammatory molecule HO1 (Fig. 3c) (p < 0.05 at 50 µM). Furthermore, pre-incubation of lung epithelial cells, fibroblasts or endothelial cells with esomeprazole substantially inhibited bleomycin-induced inflammatory response, as well as markers of fibrotic response including components of the TGFβ/MMP pathway (Fig. 4a–c). In addition, the expression of HO1 was increased in each of these lung cell types (Fig. 4d–f) (p < 0.05 at 50 µM).

### Esomeprazole inhibits TGF-β-induced collagen synthesis by lung fibroblasts isolated from IPF patients

**Fig. 5:**
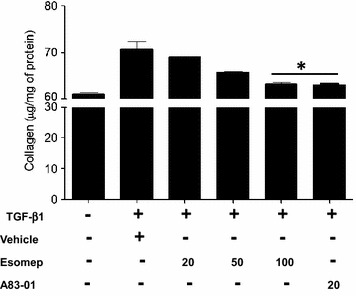
The effect of esomeprazole on soluble collagen production. Lung Fibroblasts were isolated from patients diagnosed with IPF according to International Standards. Cells were treated with vehicle, PPI (20 to 100 µM esomeprazole) or TGF-β1 inhibitor (A83-01) for 24 h. Acid soluble collagen was measured using Sircol assay. Data is Mean ± SEM from duplicate experiments. *p < 0.05 compared to vehicle.

Here, we investigated whether esomeprazole directly regulates soluble collagen production by lung fibroblasts. Primary fibroblasts were isolated from IPF patients as described and were characterized by positive staining for several myofibroblast markers (Additional file 1: Figure S2). Intriguingly, incubation of the cells with esomeprazole attenuated TGF-β-stimulated collagen release (Fig. 5).

### Esomeprazole suppresses lung inflammation and fibrosis in vivo

**Fig. 6:**
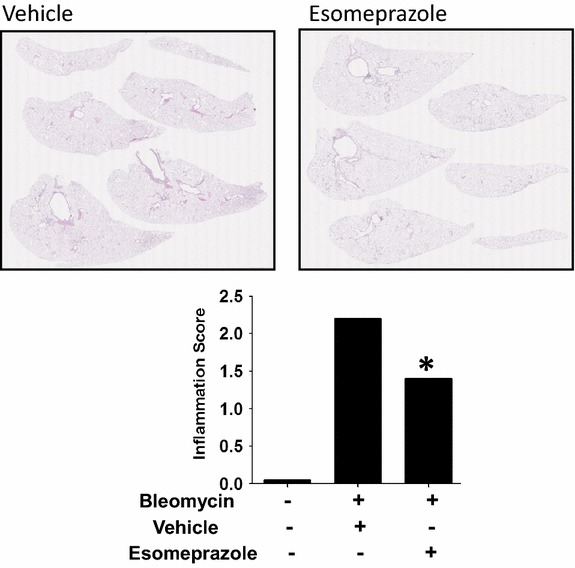
Histology of H&E stained lung sections showing suppression of inflammation by esomeprazole. Lung inflammation was induced by injuring animals with bleomycin instillation. The animals were treated with vehicle or low dose esomeprazole (prophylactic) for up to 28 days. Subsequently, lung tissues were harvested and stained with H&E to assess overall lung morphology and inflammation.* Lower panel* shows average lung inflammation score of 10 animals per group. No bleomycin sham group was included as control. *p < 0.05 compared to vehicle. Representative images are shown.

**Fig. 7:**
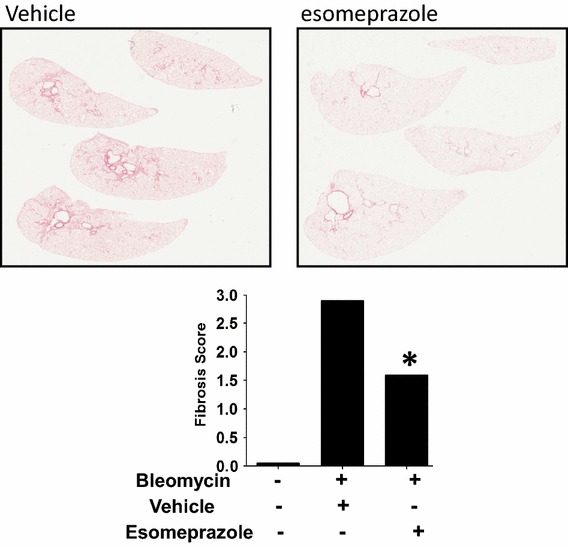
Sirius Red (collagen) stained lung sections showing the degree of accumulation of collagen fibers in lung tissue. Pulmonary fibrosis was induced by intra-tracheal instillation of bleomycin sulfate. The animals were treated with vehicle or low dose esomeprazole (prophylactic) for up to 28 days prior to harvesting and staining for collagen. Treatment with esomeprazole effectively prevented the accumulation of collagen compared to vehicle treatment as shown.* Lower panel* shows average lung fibrosis score of 10 animals per group. No bleomycin sham group was included as control. *p < 0.05 compared to vehicle. Representative images are shown.

Subsequent to the robust anti-inflammatory and anti-fibrotic property of esomeprazole demonstrated in vitro, we examined these properties in vivo in an animal model characterized by inflammation and fibrosis (i.e. the bleomycin-induced acute lung injury model). Daily administration of esomeprazole starting 2 days after the induction of lung injury by bleomycin yielded dose dependent drug levels in the plasma and lung tissue (Additional file 1: Table S1) and resulted in robust suppression of inflammation (Fig. 6) and fibrotic changes (Fig. 7) to the lungs including maintenance of normal lung tissue with no microscopically detectable lesions in 35% of the animals in the low dose esomeprazole group and in 20% of the animals that received the high dose of esomeprazole. The overall inflammation and fibrosis score is shown as Table 2. In addition, stainings of the lung tissues for the smooth muscle cell marker alpha smooth muscle actin (α-SMA) and the extracellular matrix component Collagen type 1 (Collagen 1) showed that treatment with prophylactic esomeprazole reduced their expression levels (Additional file 1: Table S2). Furthermore, there was trend towards reduced levels of soluble collagen in the BALF and lung homogenates in the prophylactic esomeprazole group (Additional file 1: Figure S3).

**Table 2:** Overall lung inflammation and fibrosis score in an animal model of bleomycin-induced lung injury

Group	Saline vehicle control	Bleomycin control	Bleomycin + esomeprazole low-dose prophylactic	Bleomycin + esomeprazole high-dose prophylactic
No. of animals examined	6	10	14	15
No. of without lung lesions	6	0	5	3
Chronic inflammation (average score)	0.0	2.2	1.4	1.6
Fibrosis (average score)	0.0	2.9	1.6	1.6

Animals were prophylactically treated with two doses of esomeprazole (30 or 300 mg/kg/day) or vehicle for up to 28 days. Subsequently, the lung tissues were harvested, stained and scored for inflammation and fibrosis.

Delayed treatment of the animals (i.e. starting 10 days after the initiation of lung injury) with esomeprazole had marginal benefit that is comparable to the efficacy of pirfenidone (Additional file 1: Figure S4). The overall score of inflammation and fibrosis for the therapeutic arm is shown in the supplemental (Additional file 1: Table S3).

**Fig. 8:**
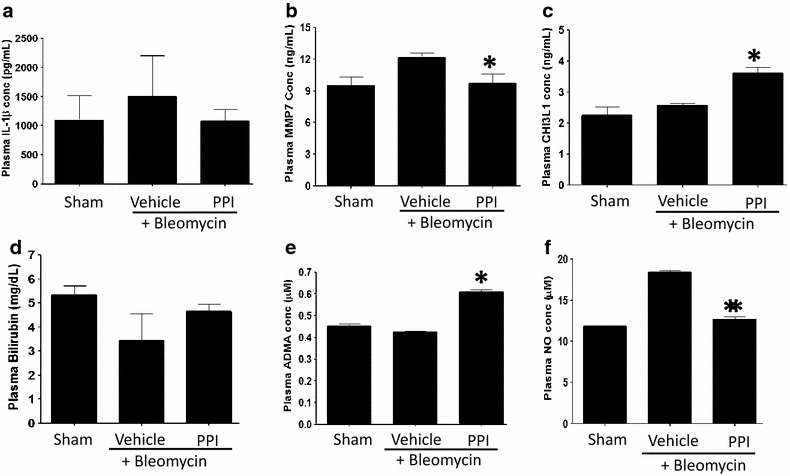
ELISA-based determination of plasma markers involved in lung pathobiology. **a** suppression of the pro-inflammatory marker IL-1β by esomeprazole; **b** inhibition of circulating MMP7 by esomeprazole; **c** increased level of the lung repair-associated protein CHI3L1 by esomeprazole; **d** higher levels of the cytoprotective and effector molecule for HO1, bilirubin, by esomeprazole treatment; **e** increased circulating level of ADMA and **f** reduced plasma NO upon esomeprazole treatment. *p < 0.05 compared to vehicle. *IL-1β* interleukin 1 beta, *MMP7* matrix metalloproteinase-7, *CHI3L1* chitinase 3-like 1, *HO1* heme oxygenase-1, *ADMA* asymmetric dimethylarginine, *NO* nitric oxide.

Furthermore, assessment of plasma for pro-inflammatory markers (e.g. IL-1β) as well as markers associated with lung remodeling (CHI3L1 and MMP7) or HO1 activity (bilirubin) revealed that prophylactic esomeprazole suppressed the level of circulating IL-1β (Fig. 8a; p = 0.579 vs vehicle) and inhibited the level of MMP7 (Fig. 8b; p = 0.0435 vs vehicle). In addition, esomeprazole enhanced the plasma levels of the lung repair-associated protein CHI3L1 (Fig. 8c; p = 0.0389 vs vehicle) and the cytoprotective molecule bilirubin (Fig. 8d; p = 0.3315 vs vehicle). In addition, based on our previous finding that PPIs inhibit DDAH enzymatic activity and block the degradation of the substrate and competitive NOS inhibitor ADMA, we evaluated the circulating levels of both ADMA and NO in the plasma of esomeprazole treated animals compared to controls. Our results indicate that PPIs inhibit DDAH activity in vivo resulting in elevated levels of ADMA (Fig. 8e; p = 0.0036 vs vehicle) and reduced NO (Fig. 8f; p = 0.0054 vs vehicle).

### Apoptotic death of lung cells is mitigated by esomeprazole

**Fig. 9:**
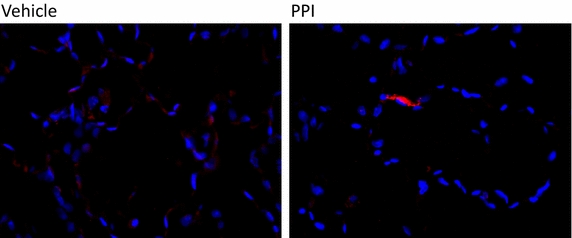
TUNEL-stained lung tissue sections showing inhibition of DNA fragmentation (shown in *red*) by the PPI esomeprazole. Animals injured by bleomycin instillation were treated with vehicle or esomeprazole (prophylactic) prior to sacrifice and staining for DNA fragmentation. Representative images are shown. The nuclei are stained with DAPI. *DAPI* 4′,6-Diamidino-2-Phenylindole.

Excessive destruction of resident cells is in part responsible for pathological remodeling of the lungs following injury. As such, protection against the death of lung epithelial cells has been proposed as a therapeutic strategy in IPF [38]. Accordingly, we evaluated the effect of esomeprazole in protecting resident lung cells from apoptosis induced by bleomycin injury. Interestingly, esomeprazole nearly abolished the apoptosis of resident lung cells as shown by reduced staining of TUNEL-based DNA fragmentation (Fig. 9, Additional file 1: Figure S5). Furthermore, double staining of the lung tissue for the pro-apoptotic marker p53 and the epithelial cell specific surfactant protein marker proSP-C showed that the apoptosis of epithelial cells is significantly reduced upon treatment with esomeprazole (Additional file 1: Figure S6).

### Esomeprazole differentially regulates several signaling pathways associated with lung inflammation and fibrosis

**Fig. 10:**
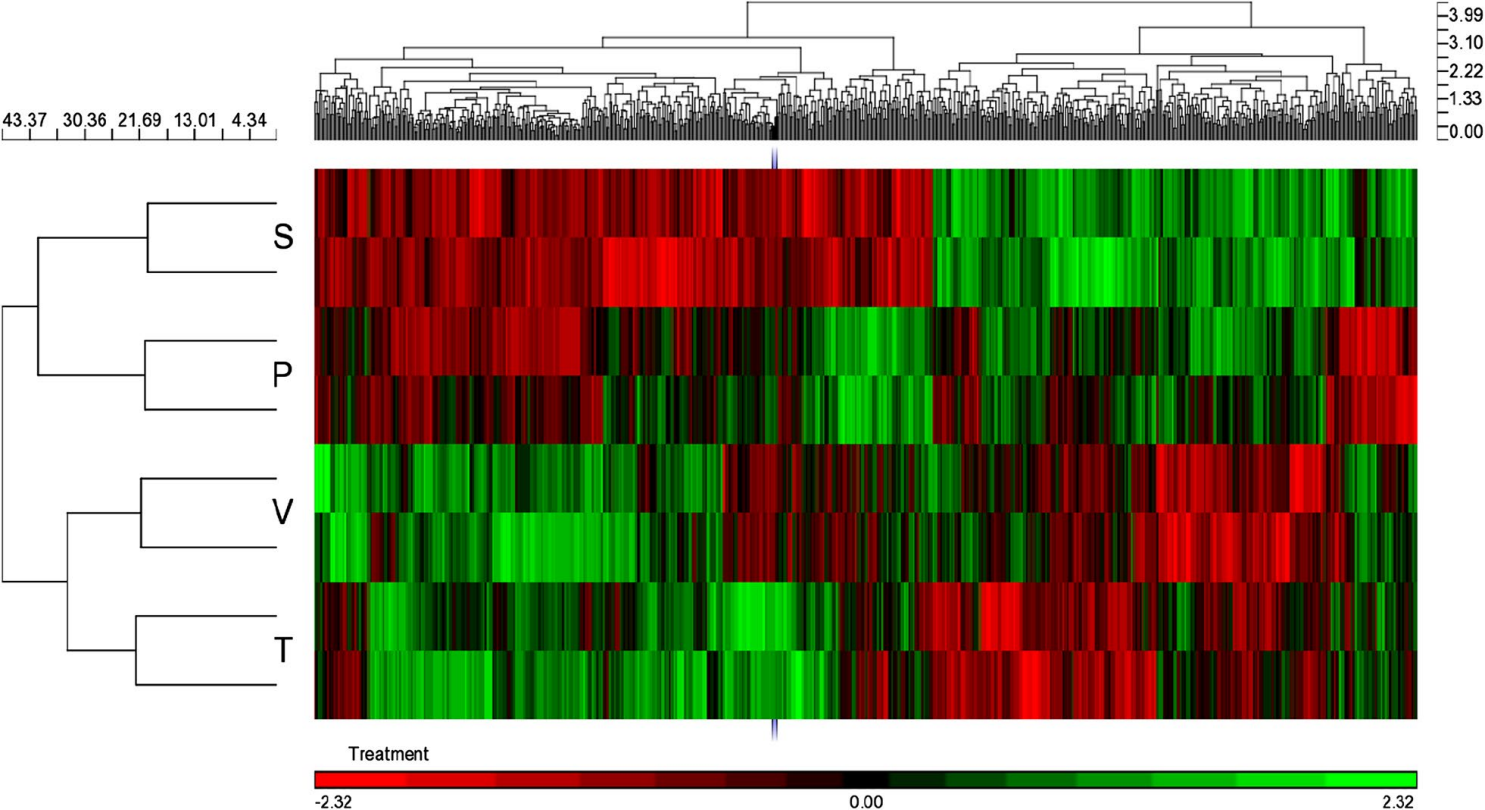
Hierarchical clustering of over 700 genes that are significantly regulated during the process of lung inflammation and fibrosis. Total RNA was extracted from the lungs of bleomycin-injured animals that received vehicle or esomeprazole (prophylactic or therapeutic course) treatment. Subsequent Genechip microarray analysis of the rat exon revealed that several transcripts are differentially regulated by esomeprazole and the prophylactic esomeprazole treatment group closely clustered with uninjured sham group. *S* sham, *P* prophylactic esomeprazole, *V* vehicle, *T* therapeutic esomeprazole.

Here, we carried out unbiased and comprehensive interrogation of signaling pathways involved in the regulation of lung inflammation and fibrosis and how these pathways are affected by esomeprazole. We conducted GeneChip array of lung tissue homogenates using a bioanalyzer as described above. Intriguingly, cluster analysis of the rat exon revealed that several signaling pathways that are known to be involved in inflammation and fibrosis including members of the Collagen family (such as Col1α2, Col3α1, Col16α1), fibronectin (FN1) and MMPs (MMP12) are regulated by esomeprazole. Interestingly, the cluster analysis of over 700 significantly regulated genes (by twofold or more at p < 0.05) indicates that prophylactic regimen of esomeprazole treatment closely resembled the gene expression signature of the uninjured sham controls (Fig. 10). Meanwhile, we also identified novel transcripts that are differentially regulated by the PPI (Additional file 1: Figure S7). One of the genes that is significantly downregulated by PPI treatment was gremlin 1 (GREM 1). Recent studies indicate that GREM1 is an endogenous inhibitor of Bone Morphogenetic Proteins (BMPs; BMP-2, BMP-4 and BMP-7) and is highly upregulated in fibrotic diseases including in IPF [39–41].

### The use of PPIs is associated with prolonged transplant-free survival in IPF

**Fig. 11:**
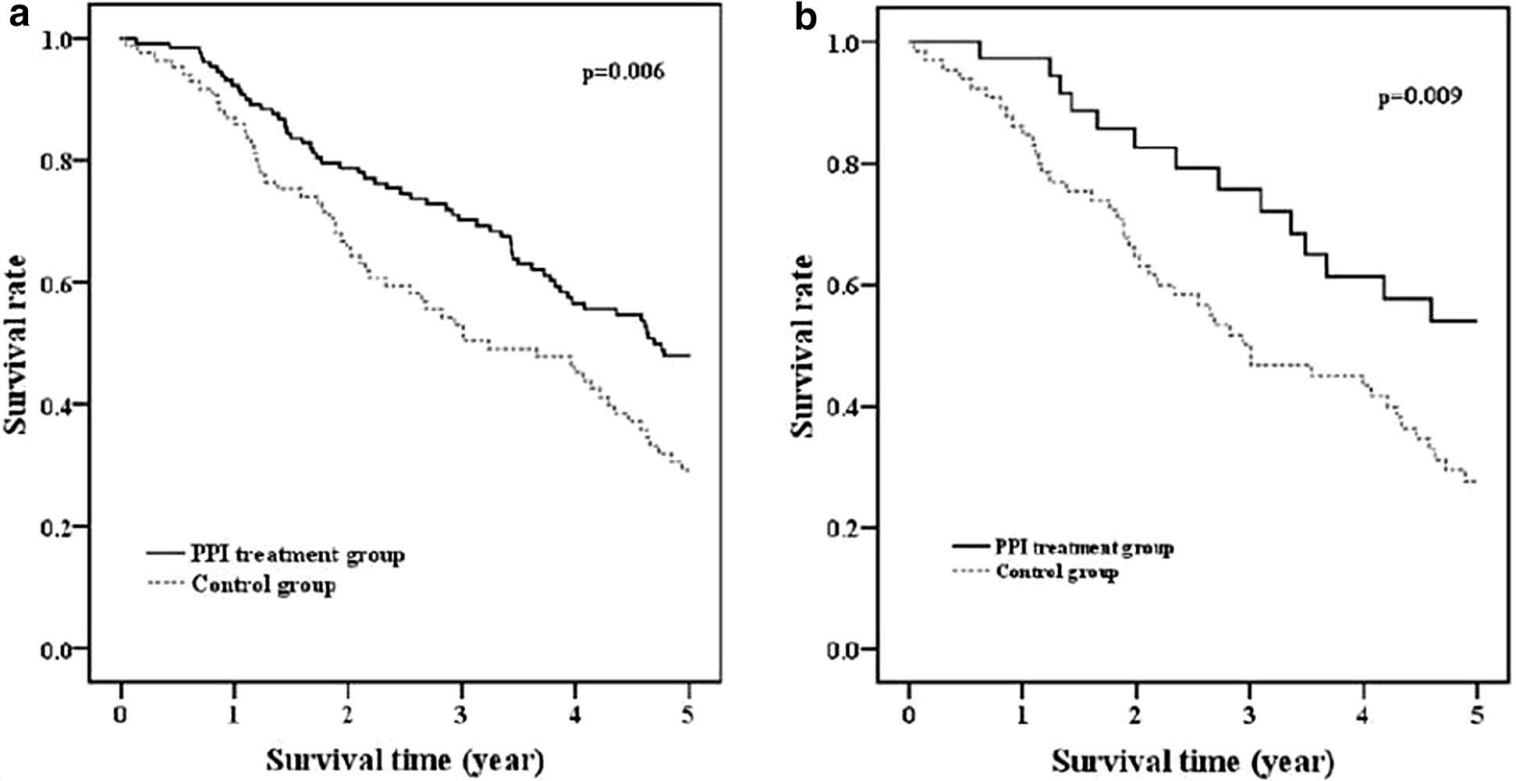
Kaplan-Meier 5-year survival plot of IPF patients. In **a** data from all 215 IPF patients is shown; In **b** only data from patients without history of gastroesophageal reflux (GER) symptoms (n = 102) is shown.

In two independent retrospective analyses of the Stanford and UAB ILD databases, long-term use of PPIs was associated with a survival benefit (data not shown). Subsequently, we merged the databases to increase our power to discriminate predictors of survival. In the merged cohort, there were no significant differences in age, body mass index (BMI), smoking history or lung function tests in those patients on PPIs for ≥12 months (n = 130) compared to the controls (n = 85). However, the use of PPIs was associated with a significant reduction in the number of patients with lung transplantation or death (p = 0.025) and a 1.4 year increase in longevity (p < 0.001) (Table 3; Fig. 11a). We also observed a similar decrease in events of lung transplantation or death and increased survival time in a subgroup of patients who had no GER-related symptoms at their initial visit but were placed on PPI therapy due to anecdotal reports of the potential benefit of PPI use in IPF (Additional file 1: Table S4; Fig. 11b). Kaplan–Meier survival plots demonstrated improved 5-year transplant-free survival in PPI-treated patients (Fig. 11). Our unadjusted and adjusted Cox regression analyses revealed that PPI treatment is an independent factor affecting transplant-free survival in IPF patients. Baseline forced vital capacity (FVC) (% predicted) (HR = 0.975, p < 0.001) and surgical lung biopsy (HR = 0.294, p < 0.001) were also independent factors affecting transplant-free survival (Additional file 1: Tables S5, S6). However, there was no significant difference between the PPI-treated group and the control group on the rate of change in FVC or diffusing capacity of the lungs for carbon monoxide (DLCO), in the 12 months following the initial pulmonary function tests (PFTs).

**Table 3:** Baseline demographics of patient population and comparison of pulmonary function tests (PFTs), lung transplantation or death, and transplant-free survival time between PPI treatment group and control group

Items	PPI treatment group (n = 130)	Control group (n = 85)	p-value
Age (years)	66 (55–73)	67 (61–76)	0.112
Male gender	81 (62.3)	54 (63.5)	0.856
Ethnicity (white, non-hispanic)	93 (71.5)	69 (81.2)	0.109
Surgical lung biopsy	61 (46.9)	35 (41.2)	0.407
BMI (kg/m^2^)	27.7 (24.5–32.5)	27.8 (25.4–30.8)	0.945
Smoking history (pack years)	6 (0–25)	5 (0–30)	0.850
Pulmonary hypertension	22 (16.9)	19 (22.4)	0.333
Lung function test			
FVC% predicted	65 (54–75)	62 (51–77)	0.440
DLCO% predicted	52 (42–65)	47 (36–57)	0.064
Patients with lung transplantation or death	77 (59.2)	63 (74.1)	0.025
Transplant-free survival (years)	3.4 (1.8–5.3)	2.0 (1.2–4.1)	0.001

Data are presented as median (25th–75th percentile) or number (percentage).

## Discussion

### Esomeprazole enhances the expression and activity of HO1 in vitro and in vivo

Several studies have demonstrated multiple functions of the proton pump inhibitors that extend beyond suppression of gastric acidity [12–14, 42–44]. For example, Becker et al. [14] reported that the PPIs omeprazole and lansoprazole possess anti-oxidant property as a result of induction of heme oxygenase 1 (HO1) expression and activity in gastric epithelial and endothelial cell lines. A subsequent mechanistic study demonstrated that lansoprazole induced HO1 expression by increasing the phosphorylation of the extracellular-signal related kinase (ERK) and Nuclear Factor-E2-related factor-2 (Nrf2) as well as inducing the nuclear translocation of Nrf2 [45]. Consistently, our study shows that esomeprazole enhances the gene and protein expression of HO1 in various primary lung cell types including bronchial/alveolar epithelial and endothelial cells exposed to bleomycin injury as well as in fibroblasts isolated from the lungs of IPF patients. In addition, we found that esomeprazole substantially upregulated the expression of HO1 in lung epithelial cells exposed to clinically relevant dosimeter of ionizing radiation suggesting cytoprotection regardless of the stimuli. Furthermore, our in vivo study shows that esomeprazole induces the expression of HO1 in the lung tissue of animals exposed to bleomycin injury. As a result, the levels of bilirubin, an effector molecule for HO1, was enhanced in the plasma of these animals (Fig. 8). Preclinical studies have shown that administration of exogenous bilirubin is protective from bleomycin-induced lung fibrosis [46].

### The HO1 pathway is involved in the regulation of lung fibrosis

As a rate limiting enzyme in the detoxification of the pro-oxidant molecule heme into equimolar concentration of three essential products, HO1 enzymatic activity is critically important for physiological regulation of cellular and tissue heme concentration [47]. As a result, overexpression of HO1 has diverse beneficial effects in various Organ Systems including the respiratory System [48]. By contrast, the expression of HO1 is reported to be decreased in alveolar macrophages derived from bronchoalveolar lavage (BAL) of IPF patients [49] and in areas of active fibrosis (i.e. fibrotic foci) [50] suggesting impaired salutary effect of HO1 in IPF. Remarkably, gene transfer-based HO1 overexpression in preclinical settings was shown to mitigate pulmonary fibrosis by suppressing aberrant lung remodeling and reducing apoptotic cell death signaling [51]. Intriguingly, our study demonstrates that comparable level of HO1 overexpression can be achieved in lung cells treated with esomeprazole (Figs. 3c, 4d–f) suggesting the therapeutic potential of the PPIs in diseases where HO1 overexpression has shown promise (outlined in Table 1 of ref [48]). Biologically, the beneficial effect associated with HO1 overexpression might be due to the enhanced release of the by-products of heme catabolism by HO1. The three effector molecules that are generated from the catalytic heme breakdown, ferrous iron (Fe^2+^), biliverdin and carbon monoxide (CO), are known to play extensive role in conferring cytoprotection. The release of Fe^2+^ stimulates synthesis of ferritin, which serves as an anti-oxidant molecule by sequestering free iron and slowing down iron-dependent redox (Fenton) reaction [52]. Biliverdin is reduced to free bilirubin; an anti-oxidant and cytoprotective molecule [53, 54]. CO, when exogenously administered, has been shown to possess multiple function including anti-oxidant, anti-apoptotic, anti-inflammatory, anti-proliferative and bronchodilator activities [52, 55]. In the lungs, transient exposure to low dose of CO (below 500 ppm) has been demonstrated to be protective from inflammation and fibrosis in the settings of acute lung injury. Zhou et al. [56] reported that inhaled CO markedly reduced the lung deposition of extracellular matrix (ECM) by ameliorating the levels of collagen I and fibronectin in a mouse model of IPF-like lung injury. Clinically, there is an ongoing Phase II interventional study evaluating the efficacy of inhaled CO in IPF disease progression [57].

### Esomeprazole is anti-proliferative and anti-apoptotic

Uncontrolled proliferation of lung fibroblasts has been reported to contribute to the excessive ECM deposition that is typically seen in the lungs of IPF patients including in areas of fibrotic foci [35]. In addition, repetitive injury and apoptosis of alveolar epithelial cells is associated with pathological regeneration of alveolar structure and leads to IPF disease progression [58]. Although the precise mechanism by which the epithelial cells in IPF lungs suffer excessive destruction is not clear, mechanistic studies have demonstrated that there is differential upregulation of the pro-apoptotic protein p53 [59] and might be responsible in driving the apoptotic response in these cells. Interestingly, earlier studies have shown that PPIs inhibit the expression of proliferation markers in cancer cells through regulation of IL-6/STAT3 pathway [60]. Our proliferation study in primary lung fibroblasts and epithelial cells cultured in the presence or absence of PPI show that esomeprazole has strong and dose dependent anti-proliferative effect (Fig. 2) suggesting the potential regulation of fibroblast-driven ECM deposition and epithelial-to-mesenchymal transition (EMT) that may result from over-proliferation of epithelial cells to supply precursor cells to become ECM-producing (myo)fibroblasts. Several findings including lineage tracing studies, although inconclusive, indicate that EMT occurs in IPF lungs [61, 62]. Meanwhile, our preclinical study shows that esomeprazole has robust anti-apoptotic effect as demonstrated by reduced DNA fragmentation (Fig. 9) and p53 immunostaining (Additional file 1: Figure S6) in the lungs of animals treated with the PPI. The IPF literature indicates that endoplasmic reticulum (ER) stress is a contributing factor to increased apoptosis of epithelial cells and its regulation has been proposed as a therapeutic strategy [63]. The inhibition of apoptotic signaling by esomeprazole may be important in reducing destruction of the resident lung cells and may allow physiological regeneration of the lung tissue exposed to stressful stimuli as in thoracic irradiation procedures. Although it is not clear how esomeprazole confers protection from programmed cell death in the lung, the downregulation of p53 and upregulation of HO1 might be responsible, at least in part, for this effect. For example, CO is known to possess potent anti-apoptotic effect [47]. Furthermore, HO1 induction might be responsible for the anti-proliferative effect of esomeprazole. Low concentration of CO has strong anti-proliferative effect and has been reported to regulate the proliferation of airway smooth muscle cells in part due to inhibition of ERK/MAPK signaling [64].

### Esomeprazole possesses anti-fibrotic activity in vitro, ex vivo and in vivo

Our cell culture study of normal lung fibroblasts exposed to bleomycin injury in the presence or absence of esomeprazole demonstrates that the gene expression of a number of pro-fibrotic markers including collagen type I and fibronectin as well as matrix metalloproteinases (MMPs) including MMP7 (matrilysin) is downregulated (Fig. 4c). In addition, our ex vivo study shows that fibroblasts isolated from the lungs of IPF patients release less soluble collagen in response to TGFβ stimulation when incubated with esomeprazole compared to vehicle control (Fig. 5). Furthermore, our biochemical assays of soluble collagen and hydroxyproline indicate that there is trend towards reduced collagen deposition in the BAL and lung tissue of bleomycin-challenged animals treated with esomeprazole (Additional file 1: Figure S3). Intriguingly, Sirius Red stained lung histology of animals challenged with bleomycin show remarkable inhibition of fibrosis by prophylactic esomeprazole (Fig. 7). In addition, administration of esomeprazole in a therapeutic regimen (starting day 10 post bleomycin challenge) shows marginal yet similar degree of reduction in fibrosis to that of pirfenidone treatment (Additional file 1: Figure S5; Table S3). It is interesting to note that IPF patients on anti-acid therapy (where the majority were on PPIs) showed reduced baseline fibrosis score compared to patients not taking the medication [8].

### Esomeprazole attenuates inflammation in primary lung cells and in vivo

The anti-inflammatory effect of PPIs has long been appreciated and their potential utility for inflammatory conditions has been discussed [13]. Although the anti-inflammatory effect of the drug has been proposed to be independent of gastric acid suppression, the exact mechanism is not clear. However, downregulation of the expression of several key mediators of inflammation including VCAM-1, TNFα, IL-1β and NFκB as well as decreased adherence of inflammatory cells to vascular wall have been reported [12, 20, 21, 65]. In the present study, we demonstrated that esomeprazole strongly diminished bleomycin- and ionizing radiation- induced elaboration of several pro-inflammatory cytokines (Figs. 3, 4). In addition, we found that the level of IL-1β in the plasma of animals subjected to bleomycin-induced lung injury was attenuated upon treatment with esomeprazole (Fig. 8). Remarkably, H&E stains of lungs from bleomycin-injured animals that received prophylactic esomeprazole treatment displayed minimal inflammation with about a third of the animals in this group showing virtually no inflammation suggesting that PPIs have potent anti-inflammatory property in vivo and may be therapeutically useful in extra-intestinal inflammatory diseases.

### Esomeprazole regulates the iNOS-DDAH pathway

About a decade ago, Genovese and colleagues [26] demonstrated that genetic or pharmacological suppression of inducible NOS (iNOS) reduces lung fibrosis in mice exposed to bleomycin injury. Recently, Pullamsetti et al. [24] confirmed the pathological role of iNOS in lung fibrosis and extended the finding by revealing co-localization of iNOS with DDAH in explanted lungs of IPF patients suggesting the interdependence of the two enzymes in the disease process. Interestingly, the pro-inflammatory/pro-fibrotic cytokine IL-1β upregulates the expression of both iNOS and DDAH [66] and a cooperative interaction between DDAH and TGFβ has been proposed [24, 67]. These interactions suggest that there may be cross-talk among inflammatory and pro-fibrotic cytokines and the iNOS/DDAH pathway. Strikingly, the work of Pullamsetti et al. revealed that the expression and activity of DDAH was increased in IPF patient lungs and pharmacological inhibition of DDAH was effective in restoring lung compliance in bleomycin-challenged mice [24]. Interestingly, we have discovered that PPIs as a class are effective inhibitors of human DDAH activity and esomeprazole is among the most potent PPIs in regulating DDAH [22] suggesting that the observed anti-fibrotic activity of esomeprazole in our preclinical study may in part be due to regulation of the iNOS-DDAH pathway.

A number of studies have shown that iNOS is induced in the setting of airway inflammation and injury [68, 69]. In IPF, gastric refluxate or other injurious stimuli are expected to release pro-inflammatory cytokines [70] that may provoke induction of iNOS. Indeed, clinical studies revealed that NO levels are elevated in BAL and lung tissue of patients with IPF [25, 71], and iNOS expression is upregulated by about threefold in explanted lungs from these patients [24]. Unlike endothelial NOS (eNOS), iNOS generates superoxide anion as well as NO which combine to produce the highly reactive peroxynitrite anion (OONO^−^). OONO^−^ forms nitrotyrosine adducts in the tissue, which interfere with normal cell signaling including proliferation and survival [72]. In addition, the nitrosative stress activates NFκB and other oxidant-sensitive transcriptional pathways to increase the expression of chemokines and adhesion molecules that augment the inflammatory response. Indeed, our data showing that the iNOS/DDAH pathway is regulated by esomeprazole is intriguing.

### Effect of PPIs on measures of lung function and survival in IPF patients

The relationship between gastroesophageal reflux (GER; an indication for which the PPIs are primarily prescribed) and lung fibrosis is well appreciated [73–76]. Studies utilizing esophageal pH monitoring have documented abnormal pH readings in the distal and proximal esophagus of a large proportion of IPF patients [10, 77, 78]. However, the precise association between GER and IPF is unclear. There are two major hypotheses regarding this relationship. The first hypothesis involves GER as a sequel of IPF. Decreased lung compliance in patients with IPF may lead to increased swings in pleural pressure causing dysfunction of the lower esophageal sphincter and eventually leading to GER [10, 79]. The alternative hypothesis revolves around chronic microaspiration of small gastric droplets either triggering acute exacerbations or leading to progressive injury and fibrosis [10, 80]. However, discordance between the high prevalence of GER (200 per 1,000) and the orphan classification of IPF (300 per 1,000,000) [81, 82], lack of clear evidence demonstrating a causal role of microaspiration in clinical pulmonary fibrosis, the grossly distinct histopathological outcomes between acid-induced lung injury in animals (mainly granulomatous inflammation) and clinical IPF (honeycomb changes without or minimal inflammation) and anatomical differences in the sites of fibrosis are findings that pose questions about a causal relationship [83, 84].

It has been assumed that the presumed benefit of PPIs in measures of lung function in IPF is due to a reduction in gastric acidity that would reduce potential lung injury due to microaspiration. Although GER commonly accompanies IPF [10, 76, 85]; and it is believed that anti-reflux strategies may benefit IPF [70], several reports have indicated the lack of direct and complete association between the progression of GER and IPF [10, 77, 80, 86–88]. Paradoxically, some IPF patients who undergo fundoplication therapy have been initially placed on PPIs and failed to suppress symptoms of reflux such as heartburn and regurgitation despite the PPIs; making them eligible for the surgical procedure (see the question and answer section of ref [70]). Thus, the use of PPIs may not provide for effective reflux control [70] and gastric reflux and microaspiration may still persist in IPF patients placed on PPIs [8, 10, 79, 89]. Furthermore, there is no evidence that the PPI-induced changes in gastric pH would reduce (lung) tissue injury in the event of microaspiration.

In light of our in vitro findings, we chose to focus our analysis of interstitial lung disease (ILD) database on the use of PPIs and potential survival benefit of IPF patients. Intriguingly, we found that the use of PPIs for 12 months or longer was associated with significantly longer transplant-free survival compared to IPF patients who did not take PPIs (Fig. 11). In principle, our finding that the use of PPIs is associated with favorable outcome in IPF is similar to what has been previously reported in the literature. Two retrospective studies have suggested an association between PPI use and improved survival [7, 8]. Moreover, deterioration in lung function has been correlated with poor adherence to PPI therapy [7]. In a case series of 4 IPF patients, Raghu et al. [7] observed clinical improvement in IPF patients on PPI therapy. Recently, Lee and colleagues [8] conducted a retrospective analysis of 204 IPF patients from two ILD databases. Ninety-eight (98) of their patients were on some form of pharmacological anti-reflux therapy as follows: PPIs = 84, H_2_-blockers = 12 and combined PPI and H_2_-blocker = 2. The use of anti-reflux medications (composed of 87% of patients taking PPIs) was associated with longer survival. In our study, we also observed survival benefit including in patients that did not have any history of GER or GER-related symptoms (Fig. 11b; Additional file 1: Table S4). In light of this, it is interesting to note that esomeprazole regulated the gene expression and plasma levels of MMP7 (Figs. 4, 8b) since clinical studies have shown that elevated level of MMP7 (Matrilysin) is associated with increased lung fibrosis and independently predicts survival in IPF [90, 91]. Meanwhile, the reduced baseline radiologic fibrosis noted by Lee et al. [8] suggest that PPIs may possess an anti-fibrotic effect, given our present in vitro and preclinical findings, however, the retrospective nature of the clinical data does not allow such a firm conclusion. Furthermore, a recent analysis of IPF patients who participated in clinical trials and were prospectively followed by the IPF clinical research network (IPFnet) showed a slower rate of forced vital capacity (FVC) decline in IPF patients on anti-reflux therapy (of whom over 90% were on PPIs) [9]. Surprisingly, the use of anti-reflux therapy was also associated with fewer episodes of acute exacerbations compared to IPF patients who did not take these medications [9]. Intriguingly, we discovered that esomeprazole significantly upregulated the expression and rat plasma levels of chitinase 3-like 1 (CHI3L1) protein (Fig. 8). A recent study reported that the lung expression and plasma levels of endogenous CHI3L1 is reduced in IPF patients with episodes of acute exacerbation compared to IPF patients in a stable condition [92]. Therefore, if our data is translated to humans, induction of CHI3L1 by PPIs might have been responsible, at least in part, for the reported incidences of fewer acute exacerbations associated with the use of PPIs in patients with respiratory diseases including IPF [9] and chronic obstructive pulmonary disease (COPD) [43].

## Conclusions

A number of inflammatory cytokines including TNFα and IL-1β are overexpressed in preclinical models of lung fibrosis and in lung tissue from IPF patients. This overexpression is known to sustain TGFβ expression and to promote the progression of the disease [93, 94]. By contrast, inhibition of these cytokines has favorable effects on fibrotic processes in cell culture and in animal models of lung injury. The literature and our present study demonstrate that the PPIs dose-dependently inhibit a number of pro-inflammatory/pro-fibrotic cytokines. We showed that a classic PPI (esomeprazole) regulated a number of players involved in the pathogenesis of lung injury including the iNOS/DDAH pathway. The inhibition of NOS/DDAH by the PPIs is important since increased NOS activity is known to reduce the tone of the esophageal sphincter [95]. Increased DDAH activity in the sphincter would be expected to enhance NO production, with resultant relaxation of the sphincter, promoting reflux. We propose, therefore, that PPIs may exhibit pleiotropic effect in mitigating lung injury and fibrosis in IPF through presumed suppression of acid reflux and inhibition of excessive release of cytokines while promoting salutary effect of HO1 and its bioactive effector molecules. Intriguingly, our retrospective analysis of ILD databases indicate that PPI use is associated with increased longevity in IPF patients. Our preclinical work suggests that there is a plausible biological mechanism for the potential therapeutic benefit associated with the use of PPIs in IPF. It is intriguing to note that the PPI esomeprazole suppressed lung inflammation and fibrosis in species (i.e. rats) that do not naturally display GER [96]. This finding suggests that the mechanism predominantly responsible for the therapeutic effect of the PPIs in pulmonary fibrosis is less likely to be suppression of GER. This finding provokes the temptation to speculate that PPIs might be beneficial in IPF regardless of the patients’ GER status. However, this possibility needs to be evaluated in prospective clinical studies by placing IPF patients with or without GER on PPIs and objectively studying measures of lung function. Moreover, future studies need to determine the concentration of PPI that is achievable in the lungs from standard oral dosing. A proportion of our present study seems to indicate that the PPI concentration necessary to achieve optimal effect on markers of inflammation/fibrosis (about 50 µM) is higher than the plasma concentration attained from a standard oral dosing for GER (up to 14 µM) [97, 98]. However, the medical records of many of our IPF patients included in the analysis indicate that many have been chronically dosed 2–3 times higher than the standard oral dose in an attempt to control GER symptoms (heartburn, regurgitation, nausea and chest pain). This higher dosing and chronic treatment of our IPF patients for several months to years may have provided beneficial effect similar to what we observed in our cell biological and preclinical studies. Furthermore, there may be discordance between plasma and tissue levels of PPIs. Interestingly, some studies propose that PPIs can accumulate in some tissues to millimolar levels [13, 42]. Finally, future mechanistic studies are warranted to investigate the precise mechanism by which PPIs regulate lung injury. In addition, prospective and controlled clinical trials are necessary to define the role of PPIs as potential therapeutic agents for IPF as well as address the contribution of GER in the pathogenesis of IPF.

## Additional file

**Additional file 1.** In the Supplemental Material Section, additional methods on characterization of IPF lung fibroblasts by immunofluorescence staining and in vitro cell toxicity assay are included. In addition, data from characterization of IPF lung fibroblasts, quantification of soluble collagen in bronchoalveolar lavage and lung homogenates of bleomycin-challenged animals, histopathological and immunohistochemical characterization of lung tissues from bleomycin-challenged animals treated with vehicle or esomeprazole, pharmacokinetics of esomeprazole in the plasma and lung tissue of bleomycin-challenged animals as well as Cox regression analysis of transplant-free survival in IPF patients placed on PPI therapy is presented.
