# Supplementary material for: Pleiotropic effect of the proton pump inhibitor esomeprazole leading to suppression of lung inflammation and fibrosis
Source: J Transl Med. 2015 Aug 1;13:249. doi: 10.1186/s12967-015-0614-x (PMC4522053; doi:10.1186/s12967-015-0614-x)
Supplement: Supplementary file 1 — Additional file 1. In the Supplemental Material Section, additional methods on characterization of IPF lung fibroblasts by immunofluorescence staining and in vitro cell toxicity assay are included. In addition, data from characterization of IPF lung fibroblasts, quantification of soluble collagen in bronchoalveolar lavage and lung homogenates of bleomycin-challenged animals, histopathological and immunohistochemical characterization of lung tissues from bleomycin-challenged animals treated with vehicle or esomeprazole, pharmacokinetics of esomeprazole in the plasma and lung tissue of bleomycin-challenged animals as well as Cox regression analysis of transplant-free survival in IPF patients placed on PPI therapy is presented. [file 12967_2015_614_MOESM1_ESM.pdf]

Additional file 1

Pleiotropic effect of the proton pump inhibitor esomeprazole leading to suppression of lung inflammation and fibrosis

Yohannes T Ghebremariam[1,2*], John P Cooke[1], William Gerhart[3], Carol Griego[4], Jeremy Brower[4], Melanie Doyle-Eisele[4], Benjamin Moeller[4], Qingtao Zhou[5], Lawrence Ho[6], Joao de Andrade[7], Ganesh Raghu[8], Leif Peterson[9], Andreana Rivera[10], and Glenn D Rosen[6]

[1]Department of Cardiovascular Sciences, Houston Methodist Research Institute, Houston, Texas, USA
[2]Department of Cardiothoracic Surgery, Weill Cornell Medical College of Cornell University, NY, USA
[3]Altitude Pharmaceuticals, Inc, San Diego, CA, USA
[4]Lovelace Respiratory Research Institute, Albuquerque, NM, USA
[5]Department of Respiratory Medicine, Peking University Third Hospital, Beijing, China
[6]Division of Pulmonary Medicine, Stanford University, School of Medicine, Stanford, California, USA
[7]Division of Pulmonary, Allergy and Critical Care Medicine, University of Alabama at Birmingham, AL, USA
[8]Division of Pulmonary and Critical Care Medicine, Center for Interstitial Lung Disease (ILD), University of Washington, Seattle, Washington, USA
[9]Center for Biostatistics, Houston Methodist Research Institute, Houston, Texas, USA
[10]Department of Pathology and Genomic Medicine, Houston Methodist Research Institute, Houston, Texas, USA

Journal title: Journal of Translational Medicine

Article number: 614

Supplemental Methods for Additional file 1:

Figure S1: In vitro toxicity assay

As a measure of acute cytotoxicity, we studied lactate dehydrogenase (LDH)- release into conditioned media by cells treated with vehicle or various concentrations (20 to 300 µM) of the PPI esomeprazole using a commercially available colorimetric assay (Sigma). The principle of this assay is based on the reduction of nicotinamide adenine dinucleotide (NAD) into NADH. This reduction, caused by leakage of LDH as a result of compromised cell-membrane integrity, is indicated by the formation of an indicator (tetrazolium dye). The resulting colored product was measured using a plate reader at 490 nm and the values were compared for statistical significance.

Figure S2: Isolation of lung fibroblasts and immunofluorescence staining

In brief, lung biopsies were obtained, de-identified and prepared for the isolation of fibroblasts by mincing, washing and seeding in tissue culture flasks in the presence of antibiotics. Fibroblasts were isolated and expanded using standard cell culture technique. The cells were characterized by immunofluorescence staining for several markers of the mesoderm lineage including fibroblast-specific protein 1 (FSP1) (rabbit; Abcam ab41532 at 1:150), vimentin (mouse; Sigma V5255 at 1:100 dilution), collagen IV (rabbit; Abcam ab6586 at 1:100), α-smooth muscle actin (α-SMA) (mouse; Sigma A2547 at 1:400 dilution), skeletal myosin heavy chain (skeletal-MHC) (mouse; Abcam ab32330 at 1:300) and caldesmon (rabbit; Abcam ab32330 at 1:250).

Supplemental Figures & Tables

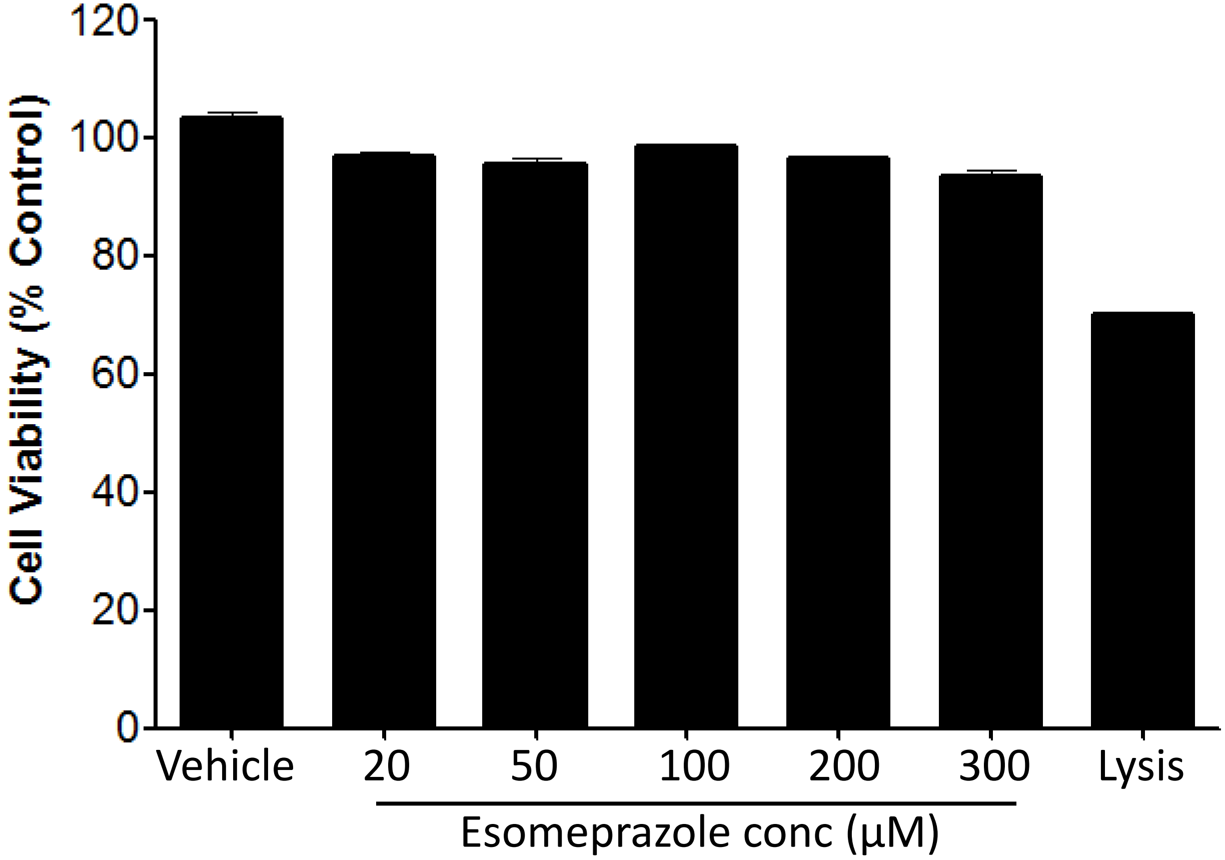

Figure S1: In vitro toxicity study. Lung fibroblasts were exposed to various concentration of PPI (20 to 300 µM esomeprazole) or vehicle and leakage of lactate dehydrogenase (LDH) was used to assess cell viability. Data is expressed as the percentage of viable cells in reference to the control treated group. Cell lysis buffer was used as a positive control.

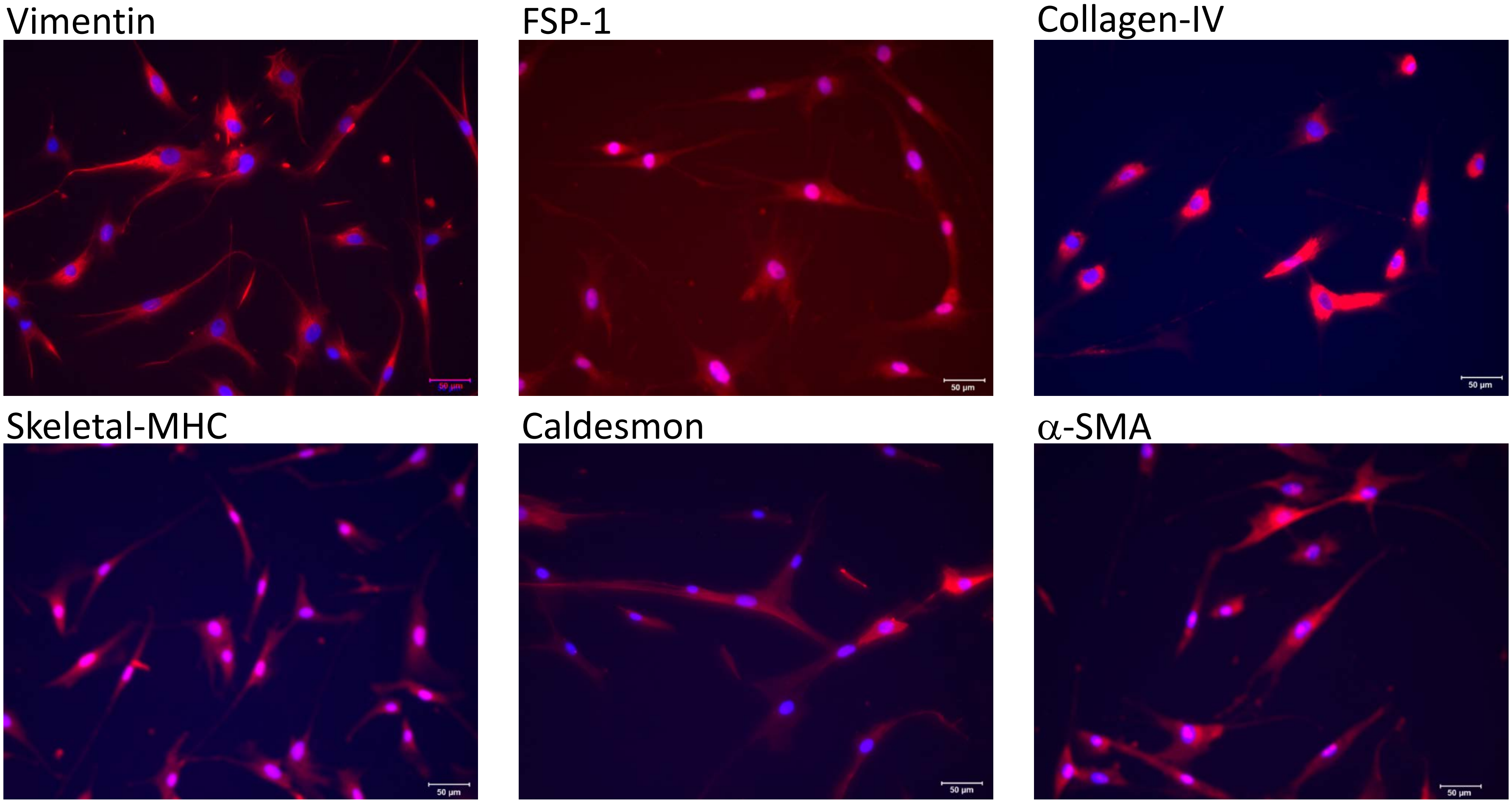

Figure S2: Immunofluorescence staining of various fibroblast and smooth muscle cell markers in lung fibroblasts isolated from IPF patients. DAPI was used to stain the nuclei and is shown in blue in each stain. DAPI = 4',6-Diamidino-2-Phenylindole. FSP-1 = Fibroblast Specific Protein 1. Scale = 50 µm

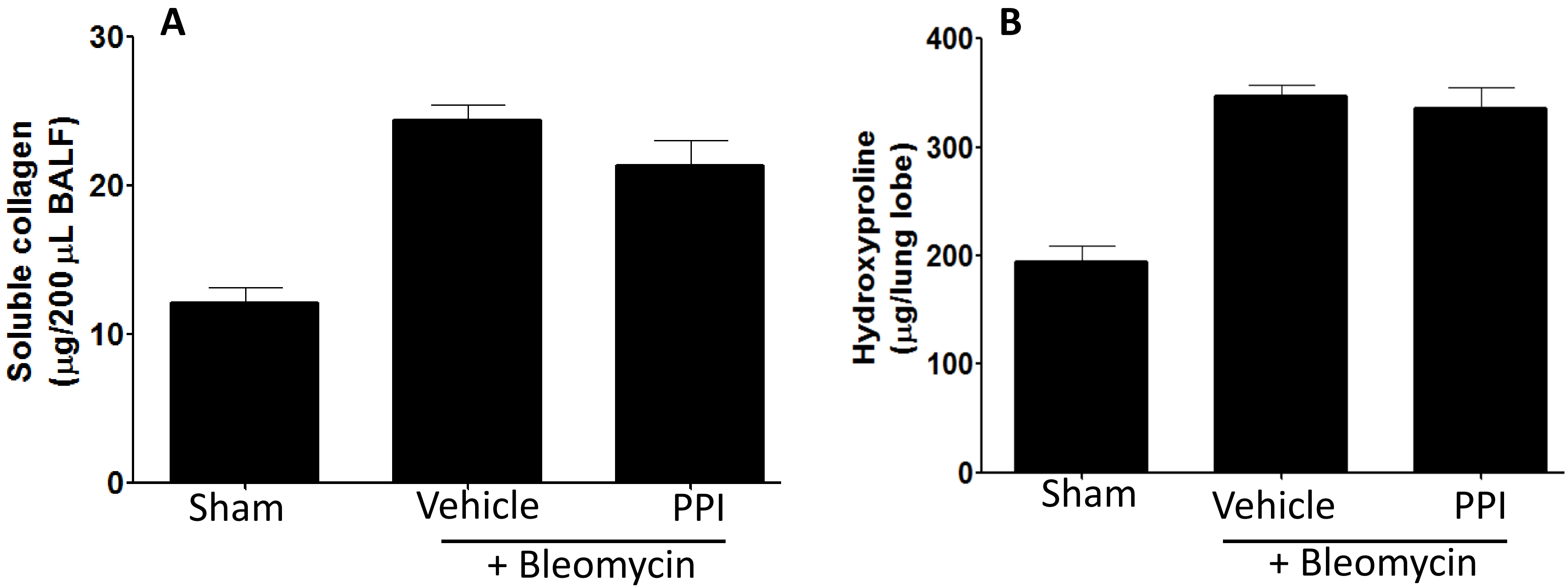

Figure S3: Estimation of soluble collagen levels in: **A)** bronchoalveolar fluid (BALF) and **B)** lung homogenates of bleomycin-injured animals that received vehicle or prophylactic PPI (esomeprazole) treatment. The levels of soluble collagen were determined by colorimetric Sircol assay following manufacturer's recommended protocol. The amount of collagen in the BALF and lung tissue samples was estimated from standard curves and was expressed as µg collagen per 200 µL BALF or µg collagen per lung lobe respectively.

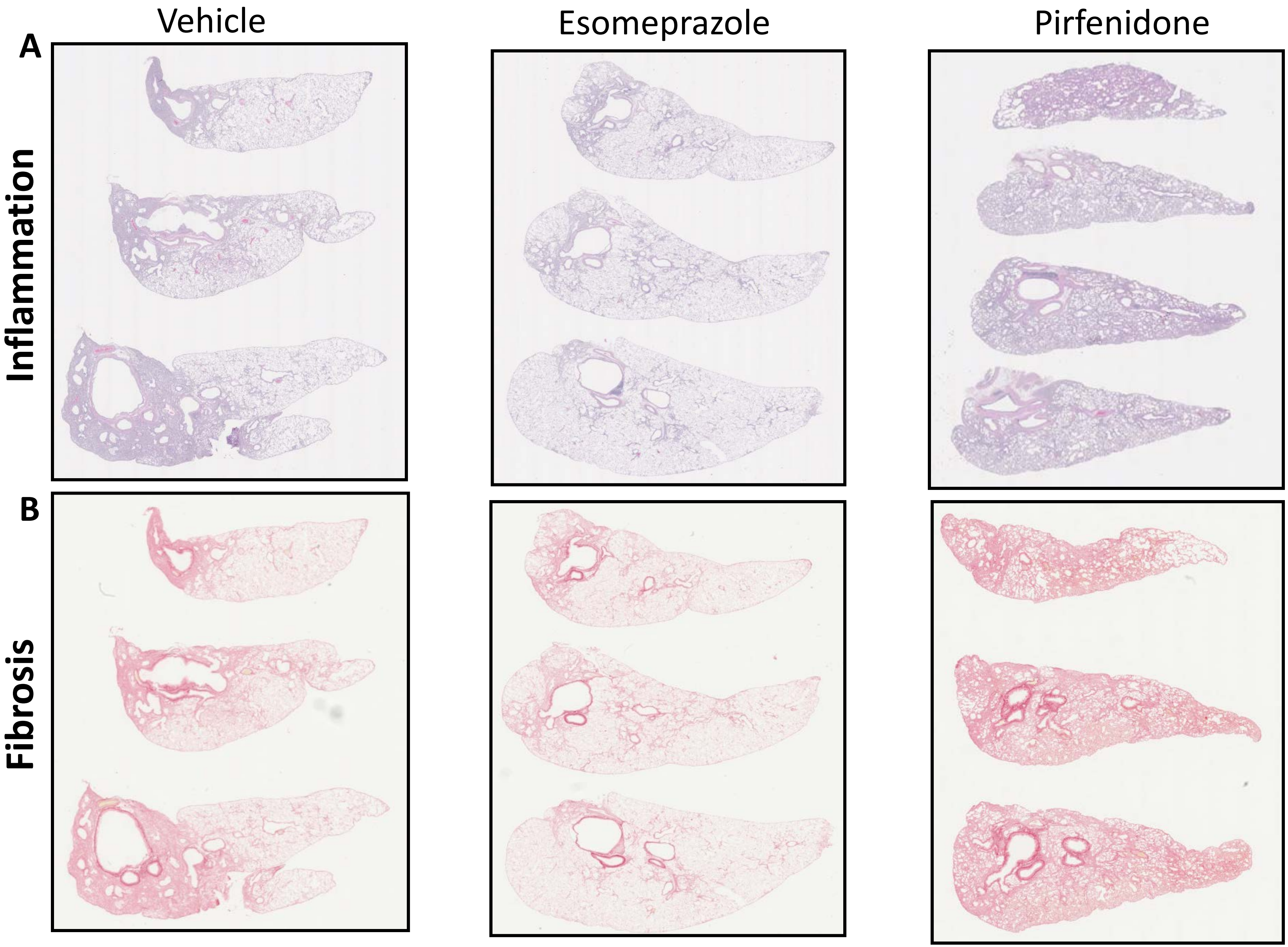

Figure S4: H&E and Sirius Red stained lung sections showing marginal effect of therapeutically administered esomeprazole on **A**) inflammation and **B**) fibrosis in a rat model of bleomycin-induced lung injury. The known anti-inflammatory/anti-fibrotic drug pirfenidone also shows minimal effect in this animal model. The animals were treated with vehicle, esomeprazole or pirfenidone for 18 days starting 10 days after inducing lung injury.

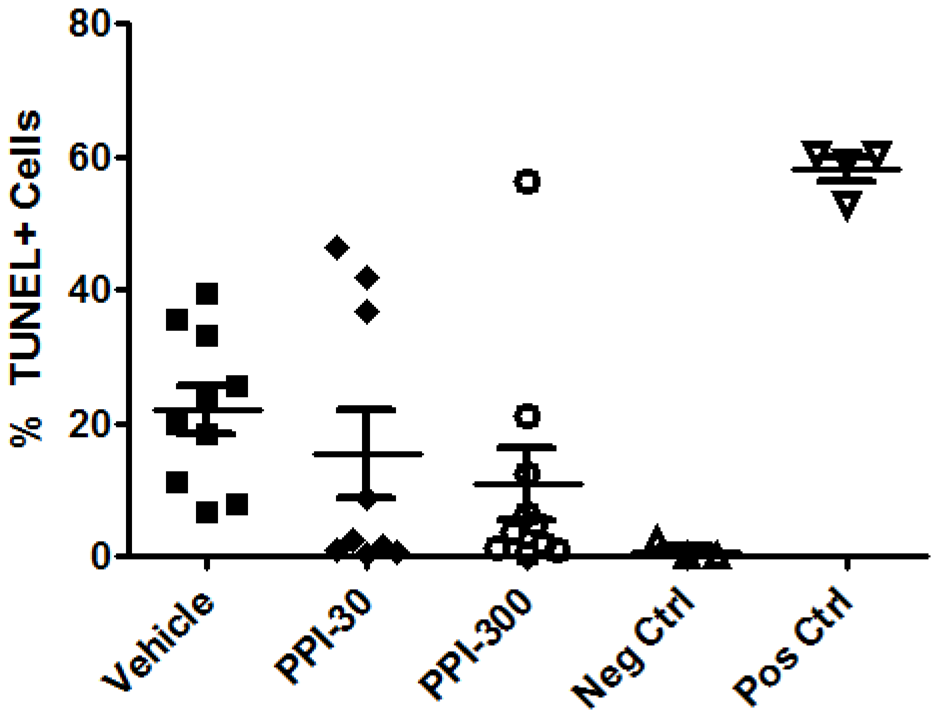

Figure S5: Overall score of DNA fragmentation in bleomycin-injured lung tissue of animals treated with vehicle or prophylactic PPI (esomeprazole at 30 mg/kg or 300 mg/kg). Multiple non-overlapping fields were scanned and average scores were recorded. Assay negative and positive controls are included as shown.

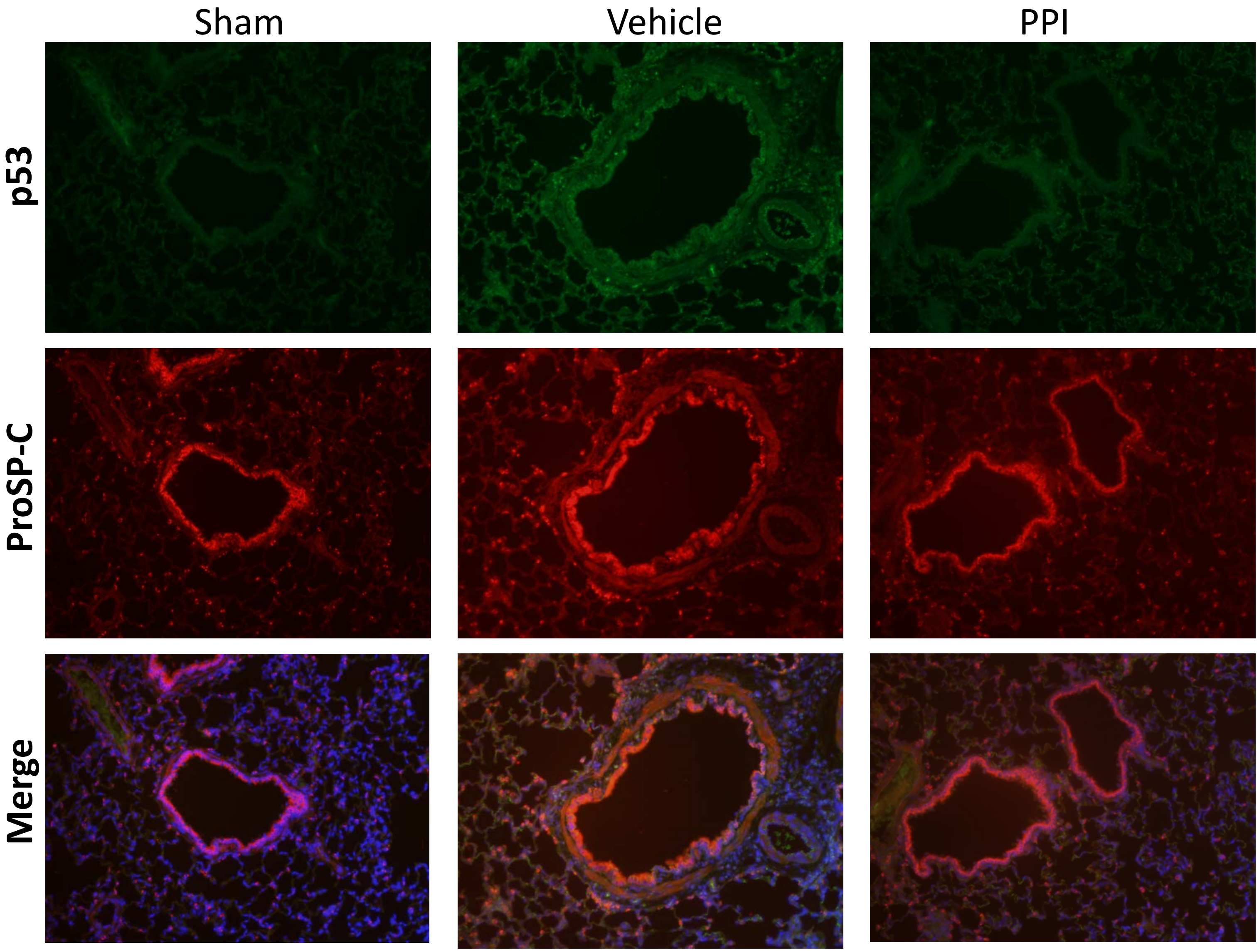

Figure S6: Immunofluorescence staining of lung tissues harvested from sham, bleomycin-injured and vehicle or bleomycin-injured and PPI (esomeprazole) treated animals. Paraffin-embedded sections were double stained for the pro-apoptotic marker p53 (green; Alexa Fluor 488 goat anti-mouse IgG) and the epithelial cell specific surfactant protein marker proSP-C (red; Alexa Fluor 594 goat anti-rabbit IgG) and show that the apoptosis of epithelial cells is reduced upon treatment with esomeprazole. The cell nuclei are shown in blue by DAPI staining.

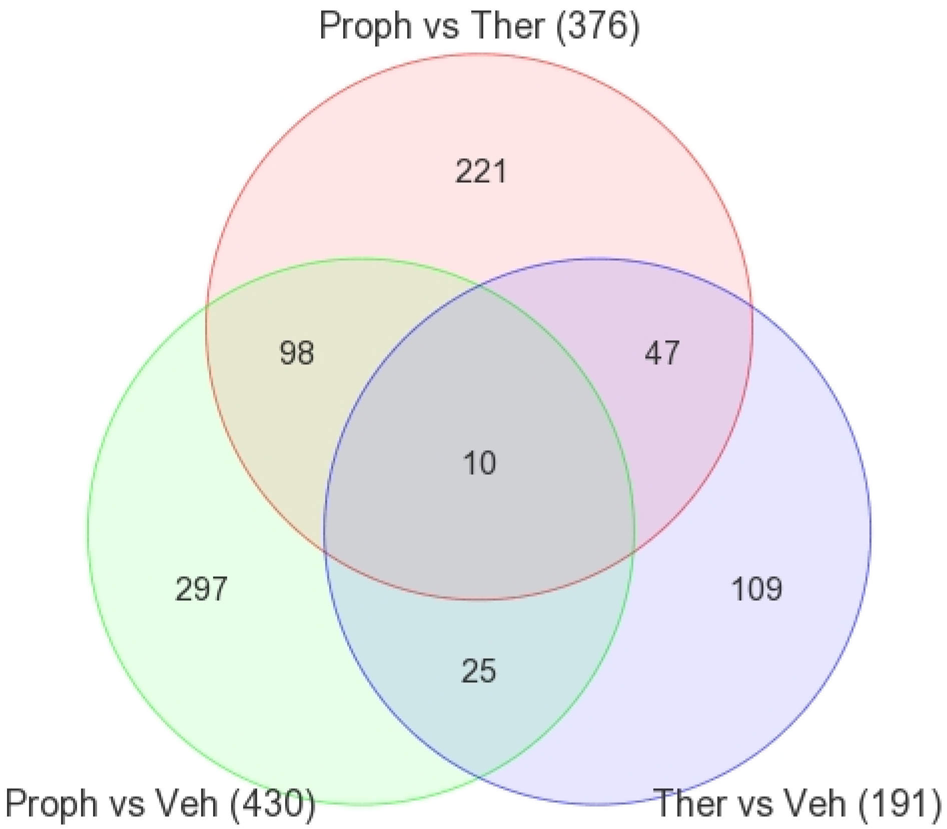

Figure S7: Venn diagram representation of microarray identified transcripts that are differentially regulated. Total RNA was extracted from the lungs of bleomycin-injured animals that received vehicle or esomeprazole treatment (in a prophylactic or therapeutic manner). Fisher's least significant difference was used for pairwise group comparisons and the total number of differentially regulated genes between any given groups is shown in brackets. The commonly regulated genes are shown by shaded areas in the figure. Veh = vehicle; Proph = prophylactic; Ther = therapeutic.

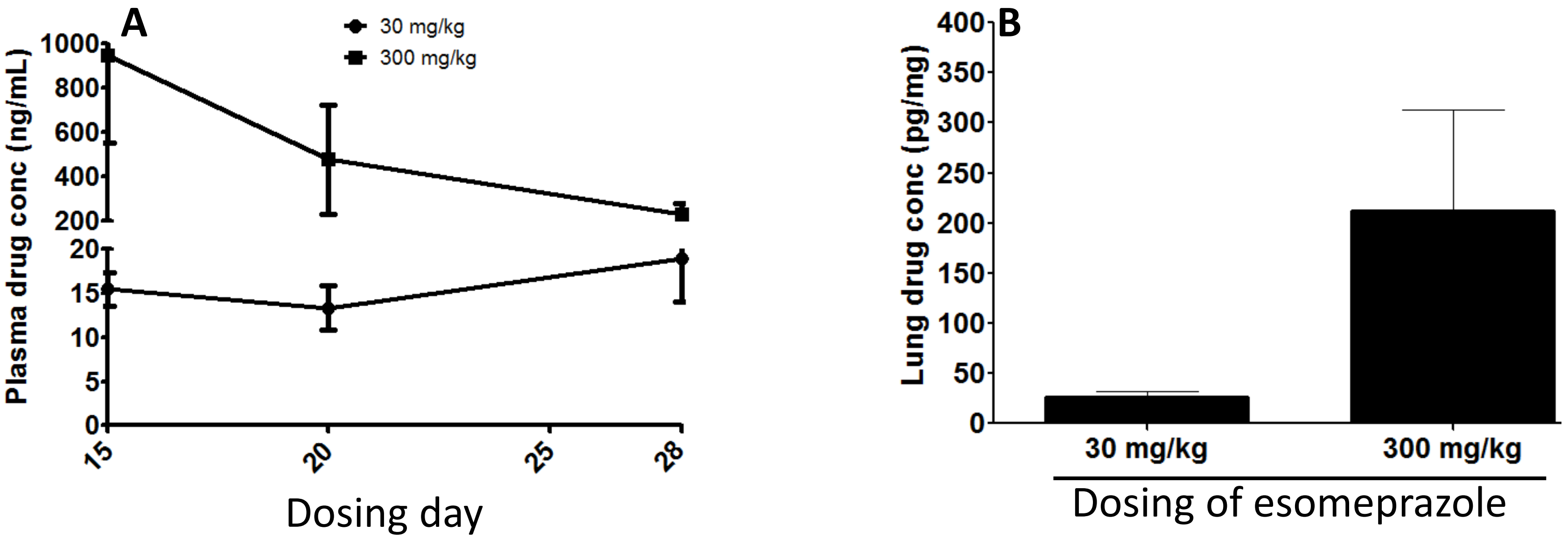

C

Parameter	Units	Dose Group 30 mg/kg	Dose Group 300 mg/kg
Tmax	hr	0.5	2
Cmax	ng/mL	52.5	7160
Tlast	hr	3	3
Clast	ng/mL	9.38	113
AUClast	hr*ng/mL	60.5	12600
AUCINF_D_obs	hr*kg*ng/mL/mg	2.68	51.7

Table S1: Determination of plasma and lung tissue drug concentration by liquid chromatography-mass spectrometry (LC-MS). **A)** Animals from the low-dose (30 mg/kg) and high-dose (300 mg/kg) of prophylactic esomeprazole (n=5 each) were used to determine the concentration of esomeprazole in plasma (on study days 15, 20 and 28) and **B)** lung tissue (at sacrifice). In **C)** Esomeprazole pharmacokinetics (PK) is shown by collecting blood at 0.5, 1, 2, and 3 hours post dosing (on study day 5). PK parameters including peak concentration (C_{max}), time to peak concentration (T_{max}) and area under the concentration/time curve (AUC) are shown.

Group	**Alpha-SMA score**	Remark	**Col Type I score**	Remark
**Vehicle**	2+ to 3+	perivascular/ bronchiole staining; also staining in areas of thickened alveolar septa	2+	perivascular/ bronchiole staining; also positive staining in areas of increased cellularity and fibrosis
**PPI**	1+ to 2+	perivascular/ bronchiole staining	1+ to 2+	perivascular/ bronchiole staining; also staining in focal areas of increased cellularity/fibrosis

Table S2: Immunohistochemical grading of bleomycin-injured and vehicle or prophylactic PPI (esomeprazole) treated rat lung tissues stained for **A)** the smooth muscle cell marker alpha smooth muscle actin (α-SMA) and **B)** the extracellular matrix component Collagen type 1 (Collagen 1) showing that treatment with prophylactic course of esomeprazole reduced their expression compared to vehicle treatment. Multiple non-overlapping fields were reviewed and representative images were scored. 1+ = minimal staining; 2+ = moderate staining; 3+ = intense staining.

Group	Bleomycin Control	Bleomycin + Esomeprazole low-dose Therapeutic	Bleomycin + Esomeprazole high-dose Therapeutic	Bleomycin + Pirfenidone
# animals examined	10	10	8	9
# without lung lesions	0	0	0	0
Chronic inflammation (average score)	2.2	2.5	2.1	2.0
Fibrosis (average score)	2.9	3.0	2.6	2.6

Table S3: Overall lung inflammation and fibrosis score in an animal model of bleomycin-induced lung injury. Animals were therapeutically treated with two doses of esomeprazole (30 mg/kg/day or 300 mg/kg/day), vehicle or pirfenidone for up to 18 days. Subsequently, the lung tissues were harvested, stained and scored for inflammation and fibrosis as shown.

Items	PPI treatment group (n=37)	Control group (n=65)	p-value
**Age** (years)	67 (55-73)	68 (61-76)	**0.156**
**Male gender**	24 (64.9)	42 (64.6)	**0.980**
**Ethnicity** (White, non-Hispanic)	25 (67.6)	51 (78.5)	**0.225**
**Surgical lung biopsy**	21 (56.8)	25(38.5)	**0.074**
**BMI** (kg/m^2)	29.7 (24.8-34.4)	27.8 (25.4-31.3)	**0.351**
**Smoking history** (pack-years)	5 (0-21)	6 (0-30)	**0.642**
**Pulmonary hypertension**	5 (13.5)	14 (21.5)	**0.328**
**Lung function tests**			
FVC% predicted	69 (60-75)	63 (50-79)	**0.171**
DLCO% predicted	56 (46-66)	49 (35-58)	**0.110**
**Patients with lung transplantation or death**	17 (45.9)	48 (73.8)	**0.005**
**Transplant-free survival** (years)	2.7 (1.7-4.8)	1.9 (1.1-3.1)	**0.020**

Note: Data are presented as median (25th-75th percentile) or number (percentage).

Table S4: Demographics and clinical data of IPF patient subgroup without history of gastroesophageal reflux (GER)-related symptoms.

	Hazard Ratio (95% CI)	*p*-value
**Age (years)**	1.008(0.990-1.026)	0.399
**Male gender**	1.131 (0.729-1.755)	0.582
**Ethnicity** (White, non-Hispanic)	1.320 (0.788-2.212)	0.291
**Surgical lung biopsy**	0.569 (0.365-0.888)	0.013
**BMI** (kg/m^2)	1.008 (0.969-1.048)	0.699
**Smoking history** (pack-years)	0.993 (0.983-1.002)	0.116
**Pulmonary hypertension**	2.289 (0.883-5.935)	0.089
**Baseline FVC% predicted**	0.981 (0.964-0.998)	0.031
**Baseline DLCO% predicted**	1.001 (0.987-1.014)	0.942
**PPI treatment**	0.596 (0.389-0.914)	0.018

Table S5: Unadjusted Cox regression analysis of predictors of transplant-free survival time in IPF. Proton pump inhibitor (PPI) use is significantly associated with prolonged survival (p = 0.018).

	Hazard Ratio (95% CI)	*p*-value
**Surgical lung biopsy**	0.561 (0.372-0.847)	0.006
**Baseline FVC% predicted**	0.984 (0.971-0.997)	0.015
**PPI treatment**	0.578 (0.386-0.866)	0.008

Table S6: Adjusted Cox regression analysis of predictors of transplant-free survival time in IPF. Both baseline forced vital capacity (FVC) and proton pump inhibitor (PPI) use are significantly associated with prolonged survival ($p < 0.05$).
